# Investigation of somatic CNVs in brains of synucleinopathy cases using targeted *SNCA* analysis and single cell sequencing

**DOI:** 10.1186/s40478-019-0873-5

**Published:** 2019-12-23

**Authors:** Diego Perez-Rodriguez, Maria Kalyva, Melissa Leija-Salazar, Tammaryn Lashley, Maxime Tarabichi, Viorica Chelban, Steve Gentleman, Lucia Schottlaender, Hannah Franklin, George Vasmatzis, Henry Houlden, Anthony H. V. Schapira, Thomas T. Warner, Janice L. Holton, Zane Jaunmuktane, Christos Proukakis

**Affiliations:** 1 Department of Clinical and Movement Neurosciences, UCL Queen Square Institute of Neurology, London, UK; 2 Queen Square Brain Bank for Neurological disorders, UCL Queen Square Institute of Neurology, 1 Wakefield street, London, WC1N 1PJ UK; 3 The Francis Crick Institute, Midland Road 1, London, NW1 1AT UK; 4 Department of Neuromuscular Diseases, UCL Queen Square Institute of Neurology, London, UK; 5 National Hospital for Neurology and Neurosurgery, Queen Square, London, WC1N 3BG UK; 6 Department of Medicine, Imperial College London, London, UK; 7 Center for Individualized Medicine, Department of Molecular Medicine, Mayo Clinic, Rochester, MN USA

**Keywords:** Multiple system atrophy, Parkinson’s disease, Alpha-synuclein, SNCA, Somatic mutation, Single cell sequencing, Mosaicism

## Abstract

Synucleinopathies are mostly sporadic neurodegenerative disorders of partly unexplained aetiology, and include Parkinson’s disease (PD) and multiple system atrophy (MSA). We have further investigated our recent finding of somatic *SNCA* (α-synuclein) copy number variants (CNVs, specifically gains) in synucleinopathies, using Fluorescent in-situ Hybridisation for *SNCA*, and single-cell whole genome sequencing for the first time in a synucleinopathy. In the cingulate cortex, mosaicism levels for *SNCA* gains were higher in MSA and PD than controls in neurons (> 2% in both diseases), and for MSA also in non-neurons. In MSA substantia nigra (SN), we noted *SNCA* gains in > 3% of dopaminergic (DA) neurons (identified by neuromelanin) and neuromelanin-negative cells, including olig2-positive oligodendroglia. Cells with CNVs were more likely to have α-synuclein inclusions, in a pattern corresponding to cell categories mostly relevant to the disease: DA neurons in Lewy-body cases, and other cells in the striatonigral degeneration-dominant MSA variant (MSA-SND). Higher mosaicism levels in SN neuromelanin-negative cells may correlate with younger onset in typical MSA-SND, and in cingulate neurons with younger death in PD. Larger sample sizes will, however, be required to confirm these putative findings. We obtained genome-wide somatic CNV profiles from 169 cells from the substantia nigra of two MSA cases, and pons and putamen of one. These showed somatic CNVs in ~ 30% of cells, with clonality and origins in segmental duplications for some. CNVs had distinct profiles based on cell type, with neurons having a mix of gains and losses, and other cells having almost exclusively gains, although control data sets will be required to determine possible disease relevance. We propose that somatic *SNCA* CNVs may contribute to the aetiology and pathogenesis of synucleinopathies, and that genome-wide somatic CNVs in MSA brain merit further study.

## Introduction

Synucleinopathies are mostly sporadic neurodegenerative diseases characterised by aggregation of the α-synuclein protein, with a wide range of pathological and clinical features, and differential selective vulnerability of cell types and brain regions [2]. They include Parkinson’s disease (PD), the closely related dementia with Lewy Bodies (DLB), and multiple system atrophy (MSA). PD and DLB are characterised by predominantly neuronal α-synuclein inclusions, with characteristic Lewy Bodies (LBs), which can also be found in apparently healthy individuals (sometimes referred to as incidental Lewy body disease, ILBD), possibly representing pre-clinical PD [20]). PD has distinct subtypes [31], and may have a multifocal onset [25]. The pathology of PD extends well beyond the substantia nigra (SN), although the defining motor features are largely due to dopaminergic (DA) neuron dysfunction and loss in the SN pars compacta. Most, but not all, patients conform to the Braak staging system [43], and spread of pathology appears dependent on connectivity and differential neuronal vulnerability [40]. The key pathological feature in MSA is glial cytoplasmic inclusions (GCIs) [44], although neuronal inclusions also occur [22], and abundant oligomer deposition is seen in neurons and oligodendrocytes [102]. α-Synuclein is likely central to the pathogenesis [80], and the aggregating α-synuclein in oligodendrocytes may be a mix of endogenously synthesised, and transferred from neurons [45, 69, 96]. MSA can broadly be classified pathologically into the predominant types of striatonigral degeneration (SND), olivopontocerebellar atrophy (OPCA), and mixed pathology, with approximately equal frequencies [75]. The clinical phenotype is determined by the pathology distribution, with SND resulting in a parkinsonian phenotype (MSA-P), and OPCA in a cerebellar [88].

Heritability for Parkinson’s disease is ~ 22% [8], and for MSA < 7% [30]. Rare inherited *SNCA* mutations, most often copy number variations (CNVs), lead to PD, often with prominent dementia, but patients frequently also have MSA features, with prominent GCIs [33, 48, 52, 86]. The CNVs are gains (duplication or triplications), leading to increased mRNA levels [77], with severity dependent on gene dosage [10]. Several other genes are implicated in PD, with either Mendelian or multifactorial aetiology [41]. *SNCA* (and other) mutations are, however, very rare in DNA derived from peripheral blood mononuclear cells of sporadic PD patients. In MSA, *COQ2* mutations may have a role only in certain populations, but there are no clear associations with other genes [47]. The magnitude of the effect of known environmental risk factors is unclear [18]. There is therefore a clear need to search for additional aetiological factors of sporadic synucleinopathies. DNA mutations also occur post-zygotically, in development or ageing. These are termed somatic, and lead to mosaicism, the presence of cells with genetic differences in an organism [122]. Mosaicism in healthy and diseased human brain is increasingly recognised, with evidence for a role in neuropsychiatric and neurodevelopmental conditions and neurodegeneration [23, 58, 71, 83, 99, 105, 113]. This arises from a wide range of somatic mutation types, including CNV and other structural variants, single nucleotide variants (SNV), and transposable element insertions. Somatic CNVs have been repeatedly reported in normal brain using sequencing of single neurons [14, 19, 38, 50, 70]. Somatic mutations may have a role in sporadic synucleinopathies [91]. If restricted to the neuroectodermal lineage, they would be undetectable in other tissues. Somatic mutations in *SNCA* or other relevant genes could lead to pathology directly if present in adequate numbers of cells of a particular region / type, or increase risk together with other factors. Even low levels of somatic mutations could be relevant, if they led to α-synuclein aggregation (or other dysfunction) in neurons carrying them, acting as a “seed” from which α-pathology spreads, and / or making the neurons carrying them vulnerable [58]. The wide variability of synucleinopathies could be partly determined by the distribution of relevant somatic mutations. Mutations in the oligodendrocyte lineage in particular could contribute to MSA. A common shared early developmental origin for neuronal and oligodendrocyte somatic mutations is possible as the “radial glia” progenitors generate both types [104], and such cells have also now been described in the SN [55].

We previously reported the results of Fluorescent in situ Hybridisation (FISH) for *SNCA* in the SN, where we found *SNCA* gains, more common in DA neurons in PD than controls, although the highest levels of DA neuron mosaicism were in two MSA cases [79]. To investigate this further, we have now studied additional brain regions, mostly the cingulate cortex, in PD, MSA and controls, and the MSA SN in more detail, by FISH, and performed single cell whole genome sequencing (WGS) in two MSA brains for genome-wide somatic CNV detection for the first time in a synucleinopathy. We present evidence of disease-related mosaicism in the cingulate gyrus, where neuronal CNVs correlated negatively with age of death in PD. We demonstrate mosaicism due to *SNCA* CNVs (gains) in the MSA SN DA neurons (identified by neuromelanin) and other cells, with higher levels in olig2-immunoreactive cells. Notably, in MSA SN, CNVs in neuromelanin-negative SN cells were associated with the presence of α-synuclein inclusions in some cells, while in LB cases this was seen for DA neurons. Finally, we report widespread genome-wide CNVs in MSA brain single neurons and non-neuronal cells, with distinct patterns. Our previous and current results suggest a possible role of somatic CNVs of *SNCA* in MSA, and synucleinopathies in general, and raise the question of the possible contribution of other somatic CNVs, which requires further investigation.

## Methods

### Human tissue

Fresh frozen and formalin-fixed paraffin-embedded (FFPE) brain tissue samples from controls and Parkinson’s disease were provided by the Parkinson’s UK tissue bank and the Queen Square Brain Bank. The latter also provided fresh frozen and FFPE brain samples from MSA cases. This study has been approved by the National Research Ethics service London – Hampstead (10/H0729/21) in addition to approval from brain tissue banks by the UK National Research Ethics Service (07/MRE09/72). All donors had given informed consent for the use of the brains in research. In total, 26 Parkinson’s disease patients, 15 MSA patients, 5 ILBD and 18 controls were used in this study. As the MSA SN was one of our main interests, we only selected MSA cases with clear pathological involvement of this region. Demographics, and a summary of all experiments performed on each sample, are shown in Additional file 1: Table S1. α-Synuclein pathology had been excluded in all controls by immunohistochemistry (IHC). Review of pathology sections was carried out by a neuropathologist (JLH, SG, or ZJ) when required. GCIs were scored semiquantitatively, using 4-tier scoring (0-absent; 3-severe). We obtained 10 μm frozen sections from the SN at the level of the red nucleus or decussation of the superior cerebellar peduncle, putamen at the level of the anterior commissure, pons at the level of the locus coeruleus, anterior cingulate gyrus and occipital cortex. We also used the following from germline *SNCA* CNV cases as positive controls for single cell sequencing: human skin fibroblasts with *SNCA* triplication from the Iowa kindred [106], as used in our FISH validation before [79], and a slow-frozen frontal pole from a case of PD diagnosed during life with *SNCA* duplication detected by MLPA.

### Fluorescence in situ hybridisation (FISH)

We used our previously published protocol with minor modifications, using the same SureFISH (Agilent) probes as before: a custom-designed 50 kb *SNCA* probe, and a *FIPL1* probe for reference [79]. All experiments were performed blinded to disease status, and using both probes, unless otherwise stated. Sections were incubated in 0.005% pepsin pH 2.0 at 37 °C for 20 min and fixed in 1% formaldehyde for another 10 min. They were washed and dehydrated in increasing concentrations of ethanol (2 min each), denatured for 3 min at 78 °C in 70% formamide in 2x Saline Sodium Citrate (SSC) buffer, and dehydrated again in increasing concentrations of ethanol at − 20 °C. Probes were mixed following manufacturer’s instructions and denatured for 5 min at 78 °C. We hybridized the sections for 48-72 h at 37 °C. After hybridization, we washed the slides in 0.3% IGEPAL in 0.4x SSC buffer at 72 °C for 2 min and in 0.1% IGEPAL in 2x SSC buffer at RT for 1 min. Cell nuclei were counterstained with 1 μg/ml DAPI for 20 min. Slides were mounted with Prolong Gold (Life Technologies) antifade reagent and kept at 4 °C until analysis.

In the SN, DA neurons were detected by the presence of neuromelanin, and cells were classified as *neuromelanin-positive* (*NM+*) and *neuromelanin-negative* (*NM-*) accordingly. In order to analyse *SNCA* CNVs in other specific cell types, and to correlate their presence with α-synuclein inclusions in the same cells, we combined FISH with IHC. To do so, after hybridization and washing, slides were blocked with 10% goat serum and 0.2% Triton X-100 in 50 mM phosphate buffered saline pH 7.4 (PBS), incubated with the primary antibody diluted in PBS ON at 4 °C, and washed and incubated with 4 μg/ml of corresponding secondary antibody conjugated with Alexa-647 or Alexa-488 (Life Technologies). Nuclei were counterstained for 20 min with 1 μg/ml of DAPI, and slides were mounted with Prolong Gold. As the pepsin incubation step in FISH may interfere with antibody staining, pepsin concentration and incubation times, and antibody concentration, were optimised for each reaction. To detect α-synuclein, we used the following antibodies after 20 min of 0.005% pepsin incubation: a mouse monoclonal (211 Santa Cruz Biotechnology, ref. sc-12,767) at 2 μg/ml when combining IHC with *SNCA* FISH and olig2 IHC, a rabbit monoclonal (MJFR1, Abcam, ref. Ab138501) at 1 μg/ml for two-colour FISH and IHC in the pons, and a rabbit polyclonal (C20 Santa Cruz Biotechnology, ref. sc-7011-R) for two-colour FISH and IHC in the SN. We did not observe differences in the staining pattern between the different antibodies. However, after pepsin treatment only cytosolic and nuclear aggregates were detected, losing Lewy neurites (where present). To detect olig2, a rabbit monoclonal antibody (EPR2673, Abcam, ref. Ab109186) was used at 1 μg/ml after 10 min of 0.0025% pepsin incubation. Finally, in NeuN experiments, we used a mouse monoclonal antibody (A60, Millipore, MAB377) and 10 min of 0.0025% pepsin incubation.

Images were obtained on a Leica epifluorescence microscope coupled to an ORCAII Digital CCD camera (Hamamatsu) and controlled by Leica Application Suite X (Leica). For each section, square dissectors of 150 × 150 μm were acquired using a 63x oil objective. Each dissector includes a z-stack of 10 images (separated 1 μm in z-axis, 10 μm total depth). To ensure unbiased representation of the whole slide, each dissector was separated 150–300 μm from the other, resulting in around 25 to 40 dissectors per slide. For each image, channels corresponding to 408 nm (DAPI), 488 nm (*FIPL1* probe or olig2), 568 nm (*SNCA*), and 647 nm (α-synuclein or NeuN) were acquired. In SN slides, a fifth channel for brightfield was included to determine the presence of neuromelanin. In anterior cingulate gyrus and occipital cortex, only the grey matter was analysed, paying special attention to obtaining images from all the different neuronal layers. In each experiment, we calculated the fraction (%) of cells containing unique gains of *SNCA* (2 or more *SNCA* copies, and 2 copies of reference probe where used), defined as “*SNCA mosaicism*”, separated by cell type as required. To avoid possible sectioning artefacts, which could lead to incomplete or multiple nuclei, and to maintain consistency with our previous work, we did not analyse cells with < 2 copies of one or both probes, or with > 2 copies of both probes. We are thus unable to call any losses, chromosome 4 aneusomy, or aneuploidy.

### Analysis of “bulk” DNA extracted from tissue homogenates

#### Exome sequencing

Exome libraries from cerebellar DNA were prepared using Illumina TruSeq or Agilent Nextera enrichment kits following the manufacturer’s recommended protocol, and sequenced on the Illumina HiSeq platform. Reads were aligned using BWA-MEM [59] to hg19. Base quality recalibration, realignment and variant calling was done using GATK HaplotypeCaller-based pipeline. Called variants were annotated in-house using Annovar [115]. These cases have previously been analysed for *LRP10* [89] and lysosomal gene mutations [90]. CNV calling was performed using XHMM [32].

#### Genome-wide analysis

DNA was extracted using phenol-chloroform to minimise GC-related bias [81]. SNP data were obtained on the Illumina Neurochip array, processed using Illumina GenomeStudio, and further analysed using HapLOH as before [81]. Mate-pair library preparation and whole genome sequencing was performed as previously [110], with analysis using the standard in-house methods with the BIMA v.3 aligner [27].

### Nuclear isolation and immunostaining

We prepared nuclear fractions from frozen brain tissue adapting published protocols [117, 118]. In brief, we homogenized tissue samples in 0.1% Triton X-100 in Nuclear Isolation Media (NIM: 25 mM KCl, 5 mM MgCl_2_, 10 mM Tris/HCl pH 8.8, 250 mM sucrose and 1 mM dithiothreitol) using a Dounce tissue grinder. After centrifugation at 1000 g for 8 min at 4 °C, pelleted nuclei were resuspended in 25% iodixanol (Optiprep Density Gradient Medium, Sigma) in 1:1 NIM:ODN (Optiprep Diluent for Nuclei: 150 mM KCl, 30 mM MgCl_2_, 60 mM Tris/HCl pH 8.8 and 250 mM sucrose). To separate nuclei from other cell compartments, we layered the sample onto 29% iodixanol in ODN and centrifuged it at 10,300 g for 20 min at 4 °C. The resulting pellet was resuspended in 10% goat serum in PBS. After 30 min, we added the 211 mouse monoclonal anti-α-synuclein antibody (1 μg/ml), and in selected experiments a rabbit monoclonal anti-olig2 antibody (EPR2673, Abcam, ref. Ab109186; 1 μg/ml), were added. After 1 h, nuclei were pelleted by centrifugation at 800 g at 4 °C for 10 min, washed with PBS, and pelleted again. Primary antibodies were detected using goat anti-rabbit IgG and goat anti-mouse IgG antibodies conjugated with AlexaFluor-488 and 568 respectively (Life Technologies) at 2 μg/ml for 1 h. After incubation, nuclei were pelleted by centrifugation, resuspended in PBS, and kept at 4 °C until use. To preserve nuclear integrity, all the solutions were pre-chilled at 4 °C and supplemented with complete EDTA-free protease inhibitor cocktail (Roche). For human skin fibroblasts, we harvested a confluent 10 cm culture plate using 0.05% trypsin, pelleted the cells by centrifugation at 800 g for 5 min, and resuspended the cell pellet in NIM before proceeding to the protocol above.

### Manual isolation of single nuclei

We performed manual selection of nuclei using a CellRaft device (Cell Microsystems) mounted on a Nikon Eclipse TE300 inverted microscope coupled to a CCD camera (KERN optics). Nuclei were counterstained with 1 μg/ml DAPI. We seeded 5000 nuclei onto a 10,000-raft array pre-treated with Cell-Tak (Corning) following Cell Microsystems recommendations. Nuclei were allowed to settle for at least 4 h at 4 °C. We isolated individual nuclei of interest in 5 μl of 10 mM Tris-HCl 0.1 mM EDTA pH 8.0 (TE) buffer, and kept them at 4 °C until further use. To avoid cross-contamination between rafts, after retrieving each nucleus, the retrieval wand was sequentially washed with 100% ethanol, DNase I solution, and sterile PBS. We distinguished neuronal nuclei from others visually: 1) diameter > 12 μm, 2) low condensed chromatin, 3) clearly defined nucleolus. We used olig2 in certain experiments to detect oligodendroglia.

### Single cell whole genome amplification (WGA) and sequencing

We performed single cell whole WGA using SMARTer PicoPLEX Gold Single Cell DNA-seq Kit (Takara) and SMARTer DNA HT Dual Index Kit (Takara). Immediately after isolation, we lysed the nuclei and carried out preamplification reaction following manufacturer’s instructions. Preamplification products were cleaned with AMPure XP beads (Beckman Coulter) and amplified in a StepOne thermocycler (Applied Biosystems) using EvaGreen (Biotium) as reporter dye. Each library had a unique combination of indexes (SMARTer dual index kit). Resultant libraries were pooled (12 to 20 libraries per pool) and cleaned with AMPure XP beads (Beckman Coulter). We determined the final concentration of each pool using the Qubit dsDNA HS Assay Kit (Life Technologies), and the size using the Bioanalyzer High Sensitivity DNA kit (Agilent). Sequencing was performed on Illumina NextSeq v3 (Illumina) using single-end or paired-end 75 base configurations following manufacturer’s indications, including 20% PhiX in all cases.

### Bioinformatic analysis of single cell sequencing

The pipeline is summarised in Additional file 2: Figure S1. Fastq files were inspected using FastQC v.0.11.5 (http://www.bioinformatics.babraham.ac.uk/projects/fastqc/). We trimmed reads using Trimmomatic-v.0.36 [9] to remove the first 14 bases (Picoplex adapters), and the final 13, to obtain 48-base pair reads, for which Ginkgo is optimised [35]. Data were aligned to hg19 using bowtie2 v.2.3.4.3 [56], sorted using Samtools-v.1.6 [60], and duplicates removed using Picard v.2.18.4 [11]. Bam files were filtered using Samtools to retain only highest mapping quality reads (q 30), and converted to bed files using Bedtools v2.25.0 [92] bamtobed command. Bam files from separate runs were merged where required using samtools merge, and downsampling of reads where required was performed using Picard. Downstream analysis, including quality metrics and CNV calls, was performed using the open-source, widely used Ginkgo [35]. We defined successful sequencing of a given single cell as follows:

1. > 800,000 reads (after processing as above).
2. Confidence score ≥ 0.8 [64]. All genomic segments are expected to have an integer copy number at the single cell level (0 or 1 in a loss, 3 or more in a gain), and this indicates the extent to which the data conform to this, rather than to intermediate copy number states, which may indicate uneven amplification [70].
3. Median absolute pairwise deviation (MAPD) < 0.3. This is a measure of the “noise” due to uneven amplification between neighbouring genomic regions, and even higher values have been considered acceptable (0.4 [98] and 0.6 [3]).

We did not attempt to call CNVs on the Y chromosome. We performed additional filtering of calls to minimise the chance of false positives. We used Mixtools Version 1.1.0 [6] to fit a three Gaussian mixture model of the copy number of all called segments around 1, 2 and 3 [19]. We then determined the precise copy number value from a cumulative two-tailed probability of 1% using the centered Gaussian distribution near 2, and we only considered losses and gains with copy numbers outside these values (< 1.45 and > 2.55 respectively). We surveyed all calls, and removed CNVs which were: (a) smaller than 3 bins, (b) in a region where the sequencing coverage showed an apparent “wave”, rather than sharp increase or decrease (inspecting it using different bin sizes if necessary), and (c) called with borderline copy number values in multiple cells, usually around centromeres.

### CNV feature analysis

#### Gene ontology and pathway analysis

We annotated a list of genes with genomic coordinates overlapping each CNV using AnnotSV [37]. These lists of CNV-affected genes were submitted to PANTHER Version 14.1 [74] to determine if any terms were over-represented at a significance threshold of 0.05 at different annotation modules. We analysed the reactome, and the following PANTHER modules: pathway, protein class, and Gene Ontology (GO) sub-modules of molecular function, biological process, and cellular component. For the GO, we used the “slim” modules, which are carefully curated to obtain a broad view of categories and improve detection efficiency. The resulting GO terms and corresponding *p*-values were submitted to REViGO [108] to aid visualization via downloadable plotting scripts [19].

#### Enrichment analysis

We used bedtools v2.25.0 [92] to test for enrichment of CNV boundaries (excluding aneuploidies), computing the co-ordinates of a 1 Mb region, with 0.5 Mb on either side of each boundary of a given CNV, telomeres, fragile sites, and segmental duplications. We used bedtools random to obtain 3000 1 Mb regions. We obtained co-ordinates of segmental duplications from the UCSC Genome Browser (Segmental Dups track), and of fragile sites from a previous study [34], lifted over to hg19. Z-scores for each CNV were calculated as described [50], with values > 1.96 indicating enrichment, and < − 1.96 depletion (significance level 0.05).

### Statistical analysis

Statistical analyses were performed with GraphPad Prism v.8.3 except as stated below. We analysed data for normality by the D’Agostino and Pearson omnibus; where this could not be demonstrated, non-parametric statistical tests were used. In an exploratory analysis, we calculated correlations using the Spearman method, as we did not assume a linear relationship between the variables, and we cannot assume a Gaussian distribution in the population from our modest sample sizes. Fisher’s exact test was used for 2 × 2 tables, unless the sample size was very large in which case chi-square with Yates’ correction was used.﻿ Relative risk (RR) and odds ratio (OR), with 95% confidence intervals (CI), are provided where appropriate. All *p*-values are two-sided where applicable, and all are nominal, unless otherwise stated. Confidence scores of cells and statistical tests of the CNV enrichment analysis were calculated using the R software environment [93].

## Results

### *SNCA* gains in the cingulate cortex are more frequent in synucleinopathies than controls

**Fig. 1:**
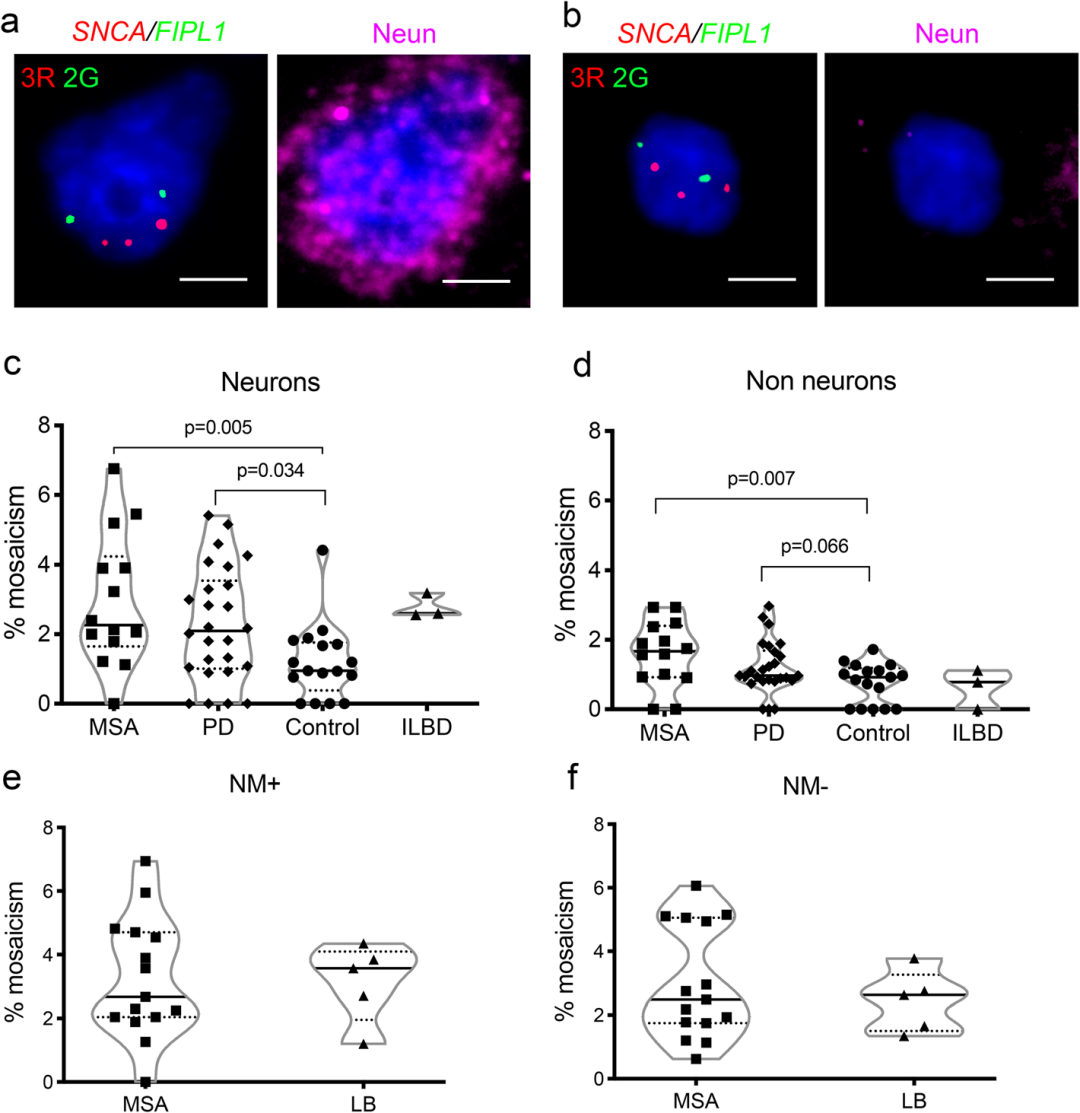
Mosaicism for *SNCA* gains. **a**, **b**. Combined FISH and NeuN IHC images of a neuron (**a**) and a non-neuronal cell (**b**) from cingulate cortex showing 3 copies of *SNCA*. Scale bar 5 μm. **c, d**. The % of mosaicism in cingulate cortex, in neurons (**c**) and non-neurons (**d**). *p* values were corrected for 2 comparisons. **e**, **f**. The % of mosaicism in the SN in NM+ cells (**e**) and NM- cells (**f**). LB cases included four ILBD and one DLB. The medians and interquartile ranges are shown in (**c**-**f**)

In our previous work, we analysed mostly the SN, and found higher mosaicism for *SNCA* CNVs (gains) in NM+ cells in PD than controls. We had performed minimal analysis of regions outside the SN, detecting neuronal mosaicism in the frontal cortex of 2/4 PD cases [79]. As SN DA neurons are selectively vulnerable in synucleinopathies due to their biology [109], those carrying *SNCA* gains may be particularly vulnerable, if these lead to increased protein. They could therefore be lost early in disease, reducing the level of mosaicism found at end stage disease. Somatic gains may therefore be paradoxically easier to detect in less susceptible regions. We chose to focus on the cingulate cortex, which has little neuronal loss, but extensive α-synuclein oligomer deposition in PD [97] and MSA [102]. To help identify neurons, we used NeuN IHC combined with FISH for *SNCA* and reference probes (Fig. 1a,b). We studied the anterior cingulate gyrus from 26 PD, 14 MSA, and 3 ILBD cases, and 17 controls (Table 1; Additional file 1: Table S1).

**Table 1:** Overall mosaicism findings in the cingulate cortex (CC) and SN

			Numbers of cells analysed for *SNCA* gains		Cells with *SNCA* gains			
					Overall		Per case (%)	
Disease	Region	Cell type	Total	Per case (mean, SD)	Number	%	Median	Mean (SD)
MSA	CC	Neuron	1359	97.1 (21.8)	38	2.80	2.27	2.94 (1.90)
		Non-neuron	1513	108.1 (15.3)	23	1.50	1.67	1.60 (0.95)
	SN	NM+	1282	85.5 (9.7)	41	3.20	2.67	3.26 (1.88)
		NM-	3397	226.5 (32.8)	103	3.04	2.49	3.01 (1.77)
PD	CC	Neuron	2533	97.4 (10.2)	58	2.29	2.1	2.31 (1.68)
		Non-neuron	2851	109.7 (16.5)	33	1.16	0.97	1.23 (0.77)
Other LB	CC	Neuron	249	83 (9.5)	7	2.81	2.60	2.78 (0.35)
		Non-neuron	296	98.7 (26.8)	2	0.68	0.78	0.63 (0.57)
	SN	NM+	411	82.2 (6.8)	13	3.16	3.57	3.13 (1.23)
		NM-	897	179.4 (27.7)	21	2.34	2.63	2.43 (0.96)
Control	CC	Neuron	1702	100.1 (19.7)	19	1.12	0.95	1.20 (1.08)
		Non-neuron	2028	119.3 (24.6)	16	0.79	0.92	0.76 (0.57)

The cell numbers analysed in each case are provided as a total, with the mean and SD per case. The numbers per individual case are shown in Additional file 2: Figure S2. The % mosaicism for *SNCA* gains of each disease / region / cell type is provided overall, as well as the median, mean and SD per case. “Other LB” refers to ILBD, except for one SN which was from a case of DLB

We identified *SNCA* gains in the cingulate cortex in all disease cases, except one MSA, and all but two controls. These were usually found in both neurons and non-neurons, but in a few cases only in one cell type. Interestingly, the SN from a case with no evidence of cingulate mosaicism had been analysed in our previous work [79], and had minimal mosaicism in NM- cells only (0.34%). We calculated the % mosaicism for cells with *SNCA* gains and 2 copies of the reference gene in each sample as before (Table 1; Additional file 2: Figure S2a; see also methods). We are not able to confidently detect losses due to the possibility of sectioning artefacts (see methods section). The overall level of *SNCA* gains across all cases was significantly higher in neurons in both MSA (2.80%) and PD (2.18%) than controls (1.12%; respective Fisher’s exact *p* values 0.0007, 0.0047). In non-neurons, it was significantly higher in MSA (1.50%) but not PD (0.97%) compared to controls (0.79%; respective Fisher’s exact *p* values 0.0007 and 0.2441). Similar results were obtained when comparing the median of the mosaicism levels in each disease case with controls, with neuronal levels higher than controls in both diseases (Fig. 1c), but non-neuronal levels significantly higher in MSA only (Fig. 1d). There were no significant differences between SND and mixed MSA (10 and 4 cases respectively; Additional file 3: Table S2). The ILBD cases were too few to formally compare, but all showed mosaicism (Table 1; Fig. 1c,d). We also investigated whether the level of mosaicism in the two cell types was correlated. There was an overall modest correlation with nominal significance when all samples were considered together (*r* = 0.35, *p* = 0.020). This was mostly driven by the PD cases (*r* = 0.40, *p* = 0.045; MSA *r* = − 0.10, *p* = 0.72; control *r* = 0.23, *p* = 0.38). These data have to be interpreted cautiously in view of the multiple comparisons.

### Somatic *SNCA* gains are seen in the SN in MSA and ILBD

We previously only analysed the SN from five MSA cases, along with 40 PD and 25 controls. The highest DA neuron *SNCA* mosaicism was observed in two of the MSA cases (~ 2.5–3%) [79]. We now analysed the SN in an additional 15 MSA cases (10 MSA-SND and 5 mixed; Table 1; Fig. 1e,f). Exome sequencing available in 13 of these was negative for variants in *SNCA* and *COQ2*. In parallel blinded experiments, we also analysed five LB cases (four ILBD, one DLB; Table 1; Fig. 1e,f). In some cases, we also performed IHC for α-synuclein (see later). We detected *SNCA* CNVs (gains) in all cases in at least one cell type. In MSA, the overall value across all cases was 3.20% for NM+ and 3.04% for NM- cells, although there was considerable variation (Table 1; Fig. 1e,f). The highest mosaicism levels were seen in MSA-SND cases (6.94% in NM+ in one, 6.06% in NM- in another), although overall there was no significant difference between SND and mixed subtypes (Additional file 3: Table S2). To determine whether cases with higher mosaicism in NM+ cells also had higher mosaicism in NM- cells, we compared these data for all samples, and found a nominally significant modest correlation (*r* = 0.48, *p* = 0.032). We also compared the overall mosaicism levels to the control samples from our previous study [79]. The overall % mosaicism levels across all, including the one with the highest NM+ mosaicism which had subsequently been designated ILBD, had been 0.42% in NM+ (15/3532 cells) and 0.25% in NM- (19/7611 cells). The values we now obtained for each cell type in MSA and LB cases were significantly higher than the same cell type in these controls (*p* < 0.0001 for all; chi square with Yates correction for MSA NM-, Fisher’s exact for all others).

### Investigation of the correlation of mosaicism with clinical and pathological features

**Fig. 2:**
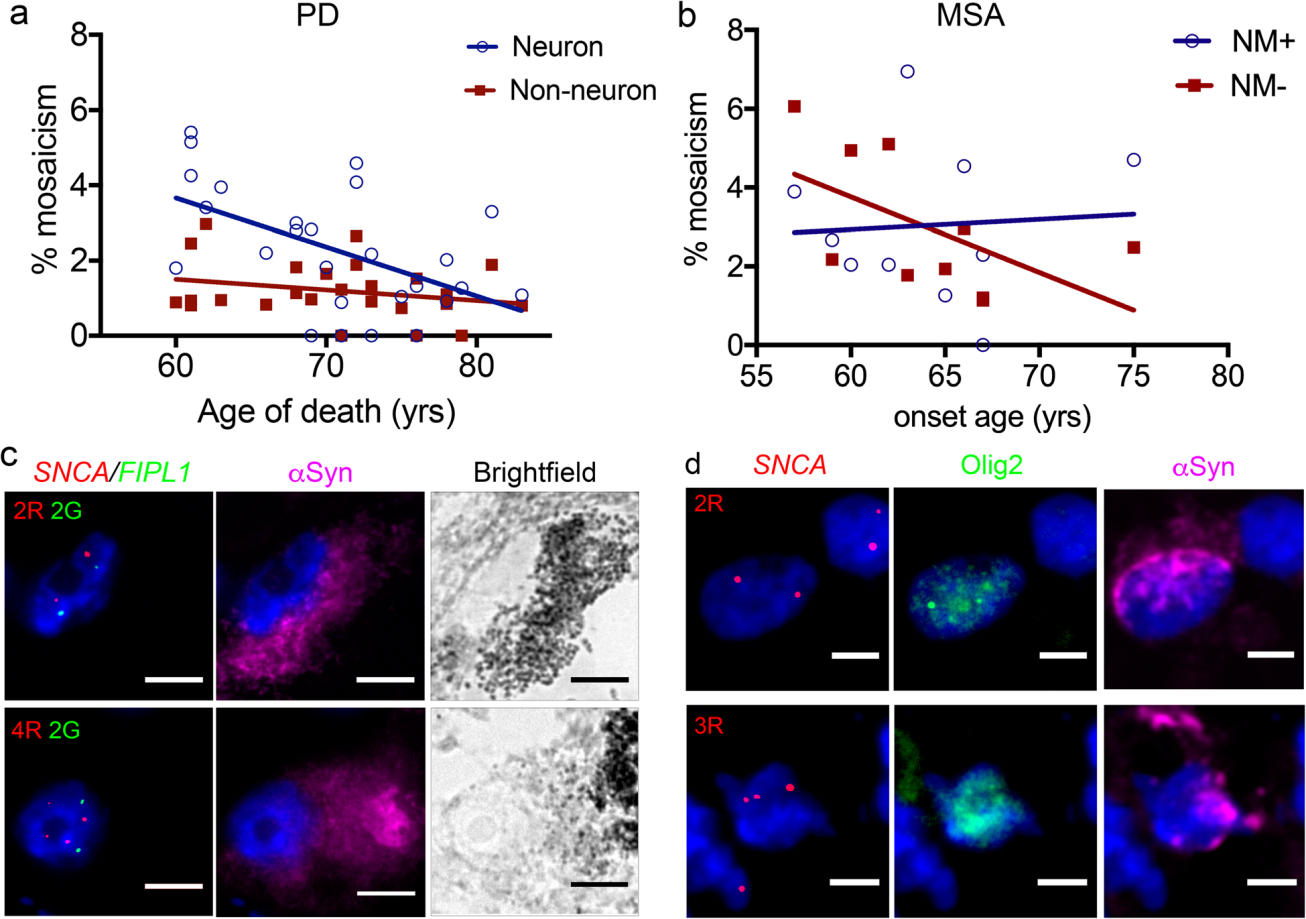
Further investigation of *SNCA* gains. **a**, **b**. Investigation of possible correlations of the level of mosaicism. Mosaicism relation to age of death in PD cingulate cortex (**a**), and to age of onset in MSA-SND (**b**). Best-fit line is shown for each cell type. Further details in text and Additional file 3: Table S3. **c**, **d** Combinations of FISH and α-synuclein IHC in DA neurons (identified by neuromelanin in brightfield) in ILBD (**c**), and in oligodendrocytes (identified by olig2) in MSA-SND (**d**). In both cases, cells with inclusions are shown, without *SNCA* gains at the top, and with gains at the bottom. Scale bar (**c**) 10 μm, (**d**) 5 μm. Note that the reference FISH probe was not used where olig2 was used

We had previously detected a negative correlation between age of onset in PD and mosaicism in the SN in NM+ cells, with higher mosaicism associated with younger onset, but no significant correlation with age of death or disease duration [79]. We investigated whether there were similar correlations in the cingulate cortex for PD or MSA, and SN for MSA-SND (Additional file 3: Table S3). In the cingulate cortex, there were no correlations of mosaicism in either cell type with onset age. In PD, there was a nominally significant modest negative correlation of neuronal mosaicism with age of death, with higher levels in those dying earlier (r = − 0.47, *p* = 0.019; Fig. 2a). No correlation with age of death was seen for non-neuronal mosaicism in PD, or either cell type in MSA. There was also no significant correlation with age of death in controls. In the MSA SN, we did not detect significant correlation of onset age, age of death, or disease duration with either cell type. Analysing MSA-SND alone also revealed no significant correlations. We noted, however, that MSA-SND cases with higher mosaicism in NM- cells tended to have younger onset (Fig. 2d; *r* = − 0.6, *p* = 0.074). Interestingly, one of these had unusually late onset at 75, more than 2 SD later than the mean in all our SND cases (64+/− 5.2), all QSBB cases (56.7 +/− 8.4) [75], and other literature (56+/− 9) [44]. The phenotype appeared relatively mild, and only “possible MSA” criteria were fulfilled in life, but there were no atypical pathological features. Excluding this patient would lead to a nominally significant correlation (*r* = − 0.78, *p* = 0.018).

We had previously not found any significant pathological correlates with mosaicism in PD SN [79]. We now investigated this for MSA in both regions, using FFPE sections from the contralateral hemisphere in a blinded manner, to compare mosaicism in each cell type with the load of GCIs. In the cingulate, we did not find any correlation of mosaicism with either cortical or subcortical GCIs (r < 0.25 and *p* > 0.5 in all; Additional file 3: Table S4). In the SN, we used sections taken at the level of the red nucleus, where available (*n* = 7, all SND). We also did not find significant correlation with mosaicism in NM+ cells (*r* = 0.60; *p* = 0.17) or NM- cells (*r* = 0.18; *p* = 0.74).

### In the substantia nigra, α-synuclein inclusions may be more common in cells with *SNCA* gains

If *SNCA* CNVs have a functional role, they may be associated with α-synuclein inclusions in the same cells, potentially in a specific cell type only in each disease. We aimed to determine whether individual cells with *SNCA* gains are more likely to have inclusions by combining FISH with IHC for α-synuclein. Our FISH pre-treatment, which includes pepsin, may remove some inclusions, and this combined analysis may only allow detection of the most robust GCIs or LBs. We performed this only in the SN, where we can use neuromelanin to distinguish cell type. We obtained data from 7 MSA-SND and 5 LB cases, where we had combined FISH for *SNCA* and reference probe with IHC for α-synuclein (Table 2). We noted that overall, inclusions were more frequent in cells with CNVs (22.1%) than without (5.7%; Fisher’s exact *p* < 0.0001). Further detailed analysis revealed an interesting pattern. In NM+ cells from LB cases, 36% of cells with gains had inclusions, against only 6% of cells without gains (RR 6.03, 95% CI 2.52–14.43; OR 8.9, 95% CI 2.40–32.6; Fisher’s exact *p* = 0.0042; Fig. 2e). No difference was seen in NM- cells, which showed inclusions in none of the cells with gains, and 2% of cells without gains (*p* = 1). In NM- cells from MSA-SND, 33% of cells with gains had inclusions, against only 8% of cells without gains (RR 4.16, 95% CI 2.44–6.52; OR 5.7, 95% CI 2.80–11.8, Fisher’s exact *p* < 0.0001). There was no such effect in NM+ cells, where the frequency of inclusions was 7.7% in cells with gains, and 6.1% in cells without gains (*p* = 0.56).

**Table 2:** Relation of *SNCA* gains and inclusions in the SN

Disease	Cell type	Cells with CNVs			Cells without CNVs		
		Total	Inclusions	No inclusions	Total	Inclusions	No inclusions
MSA-SND	NM+	13	1	12	589	36	553
	NM-	36	12	24	1185	95	1090
LB	NM+	11	4	7	398	24	374
	NM-	17	0	17	868	17	851
Total	Both	77	17	60	3040	172	2868

The most important cells in MSA may be of oligodendroglial lineage. GCIs containing α-synuclein are usually abundant in oligodendrocytes, and correlate with neuronal loss [22], although oligodendrocytes are not lost significantly [85]. Recent data also indicate the importance of oligodendrocyte precursors [45]. NM- cells in the SN are likely mostly of oligodendroglial lineage, but include other glial cells and non-DA neurons. To determine whether the NM- cells with *SNCA* gains include oligodendroglia, we combined α-synuclein IHC and FISH for *SNCA* only, with IHC for olig2, in an additional unblinded analysis of three SN samples from MSA-SND. *SNCA* gains were indeed present in cells of oligodendroglial lineage. In fact, among all NM- cells, gains were seen in 8.56% of olig2-positive cells (19/222), and 2.26% (7/310) of olig2-negative cells (RR 3.79, 95% CI 1.62–8.86; OR 4.05, 95% CI 1.67–9.82; Fisher’s exact *p* = 0.0016). Among olig2 positive cells, inclusions were seen in 31.2% of cells with CNVs (6/19), and 15.9% of cells without CNVs (33/203; Fisher’s exact *p* = 0.11; Fig. 2f). Olig2 has high expression in oligodendrocyte precursor cells [119], but may not label mature oligodendrocytes robustly [1], so we cannot exclude the possibility that olig2-negative cells with CNVs were mature oligodendrocytes. The questions of whether *SNCA* gains in MSA SN are more common in oligodendrocytes overall, and whether CNVs in oligodendrocytes are associated with inclusions in the same cells, clearly require investigation in a larger sample size.

### Preliminary analysis suggests that *SNCA* gains may also occur in other brain regions

We analysed the putamen and occipital cortex, combining FISH for *SNCA* and reference probes, with NeuN IHC, as for the cingulate cortex, in a randomly selected small number of brains (Additional file 3: Table S5). In the putamen, mosaicism in neurons and non-neurons was seen in PD (*n* = 1) and MSA (*n* = 2), and had been previously detected in another MSA case [79], but was absent in the only control studied. In the occipital cortex, we analysed 4 disease cases (2 PD, 2 MSA), including the ones where putamen was analysed. All had some evidence of mosaicism, but it was absent in neurons in one PD case, and had been absent in one previous MSA case [79]. A systematic comparison of these regions will clearly be of interest.

In MSA, neuronal nuclear inclusions can occur in certain regions [22]. We selected three MSA cases with frequent neuronal nuclear inclusions in the pontine base nuclei, and performed FISH (unblinded), combined with IHC for α-synuclein to determine if inclusions are more frequent in cells with CNVs. Mosaicism was present in all (Additional file 2: Figure S3; Additional file 3: Table S5). Non-neurons were more likely to have inclusions if they had CNVs (3/5), than if they did not (20/174; RR 5.22, 95% CI 1.87–9.80; OR 11.55, 95% CI 2.19–66.39; Fisher’s exact *p* = 0.0158). This is similar to the situation for NM- cells in the SN of MSA-SND cases, but as these data are from small numbers of cells from three pre-selected cases, they may not be representative. No difference was seen in neurons, with 3/8 neurons with CNVs having nuclear and/or cytoplasmic inclusions, against 18/92 with no CNVs (*p* = 0.3592).

### Genome-wide detection of CNV mosaicism requires whole genome sequencing (WGS) of single nuclei

We previously found no high-level mosaic CNVs in PD using targeted array comparative genomic hybridisation for several PD genes, and droplet digital PCR for *SNCA*, on DNA extracted from tissue homogenates [79], consistent with the low mosaicism levels seen by FISH. As MSA has lower heritability than PD, and we found similar or higher *SNCA* CNV mosaicism in MSA, we next focused on this for detection of genome-wide somatic CNVs. We first excluded high-level mosaicism in one case (MSA8) using two methods. We sequenced DNA from two brain regions (SN and cerebellum) using mate-pair whole genome sequencing (WGS), which has a large library insert size, facilitating structural variant detection based on discordant read-pairs [13]. No variants of possible interest were seen (Additional file 2: Figure S4). We also obtained SNP data from the SN, cerebellum and frontal cortex on the NeuroChip [7], a custom-designed array enriched for genes involved in neurodegenerative disease. We analysed data using HapLOH, which detects aberrant B-allele frequencies resulting from significant copy number imbalance due to mosaic gain or loss [101], and has been used successfully for tissue mosaicism detection [112], but noted no abnormalities.

We thus decided to proceed to single cell whole genome amplification (WGA) and WGS for detection of genome-wide somatic CNVs present at very low levels, or indeed confined to single cells. We aimed to correlate any CNV calls with the cell type of origin, and if possible the presence of inclusions in that cell. Due to the difficulty of isolation of intact neurons from post-mortem brain, single cell WGS is performed after preparing nuclear fractions [15, 19, 38, 70]. Antibodies to nuclear epitopes can be used to detect and isolate the nuclei of cells of a specific type, for example NeuN is routinely used for fluorescence-activated nuclear sorting (FANS) of cortical neurons [15, 19, 38, 70]. NeuN may not, however, be reliable in the SN [16, 54]. We therefore used size to differentiate neuronal nuclei, as used in a study sorting DA nuclei in the MSA SN [121]. In certain experiments in pons and putamen, we also used an antibody to olig2, to determine if a non-neuronal nucleus was likely to be oligodendroglial. We had already verified in our FISH / IHC experiments a nuclear staining pattern, as expected for this transcription factor. To detect and sequence cells with α-synuclein inclusions, antibodies to α-synuclein can be used. These would, however, only be expected to allow detection of nuclear inclusions, which can occur in MSA pons and putamen. We therefore decided to perform WGS on single cells from the SN, an affected region where we already had considerable *SNCA* FISH data, and the pons and putamen, affected regions where we had a limited amount of FISH data. To obtain a meaningful number of cells from each case, we restricted our analysis to two MSA cases. We studied the SN, pons and putamen in mixed MSA (case MSA15), and the SN in MSA-SND (case MSA10).

We used the CellRaft (Cell Microsystems) for single nucleus selection. This allows visual review, reducing the chance of doublets compared to fluorescence-activated nuclear sorting (FANS), and taking photographs which could be correlated with the individual cell WGS results. The CellRaft is a manual device mounted on an inverted microscope, with thousands of microwells which can be released individually with a magnetic wand. It has already been used for sequencing neuronal nuclei [19, 118]. After appropriate antibody staining, we seeded nuclei on an array, and isolated rafts with a single nucleus (Additional file 2: Figure S5a). We expected any staining to be clearly nuclear. We noted, however, that some nuclei, both neuronal and non-neuronal, demonstrated perinuclear or juxtanuclear α-synuclein staining (Additional file 2: Figure S5b-f). This presumably corresponded to cytoplasmic inclusions retained during the isolation. Presence of cytoplasmic membranous components has previously been noted in neuronal nuclear fractions, and attributed to contiguity of the endoplasmic reticulum and nuclear membrane [119]. It should be stressed, however, that the absence of synuclein staining does *not* necessarily indicate that the cell had no inclusions, as some inclusions may have been removed during the processing.

### Germline *SNC*A gains are detectable at the single-cell level

**Fig. 3:**
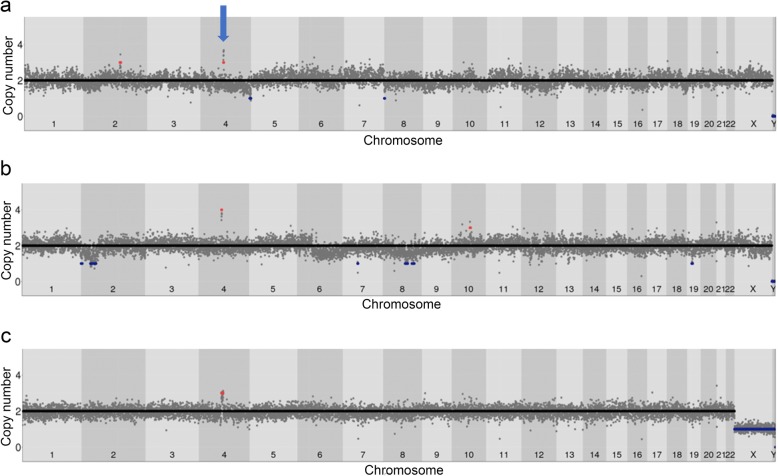
Single cell WGS profiles of cells with known germline *SNCA* CNVs (arrow). **a** Fibroblast with triplication. Note additional calls (gain in chr2, telomeric losses in chr4 and 8). **b** Lower quality fibroblast with triplication, which narrowly fails confidence score filter, and would thus not be analysed. Note increased “waviness”, and likely false positive losses in regions of a negative wave, or near centromeres. **c** Cortical neuron with duplication. Note the clear differentiation of the XY chromosome copy number in this male

To determine if our workflow could detect known megabase (Mb)-scale CNVs in single cells, we used samples with germline CNVs involving our main gene of interest, *SNCA*. We used fibroblast samples from a patient with a triplication from the Iowa kindred [106], recently sized at ~ 1.7 Mb with areas of duplication around the triplicated region [123], on which we had previously validated our *SNCA* FISH probes [79]. We isolated, amplified and sequenced two single fibroblast nuclei. The CNV was called in both, although one narrowly failed our strict confidence score threshold (Fig. 3a,b). Copy number estimation and sizing was more accurate when using 250 kb genomic intervals (“bins”) than 500 kb, and the CNV was also detected after downsampling to ~ 1 and ~ 0.5 million reads. We also attempted to sequence two neurons from a slow-frozen frontal pole of a patient with a duplication diagnosed during life, but not fully defined. One was successful, revealing a 6.7 Mb *SNCA* gain when using 250 kb bins (Fig. 3c), very similar to the 6.4 Mb CNV previously reported in an individual from the same geographical region [46]. We thus conclude that we are able to detect megabase-scale CNVs, using fewer than a million reads, at 250 kb bin size.

### Single cell WGS in MSA brain reveals CNVs, with gains predominant in non-neurons

**Fig. 4:**
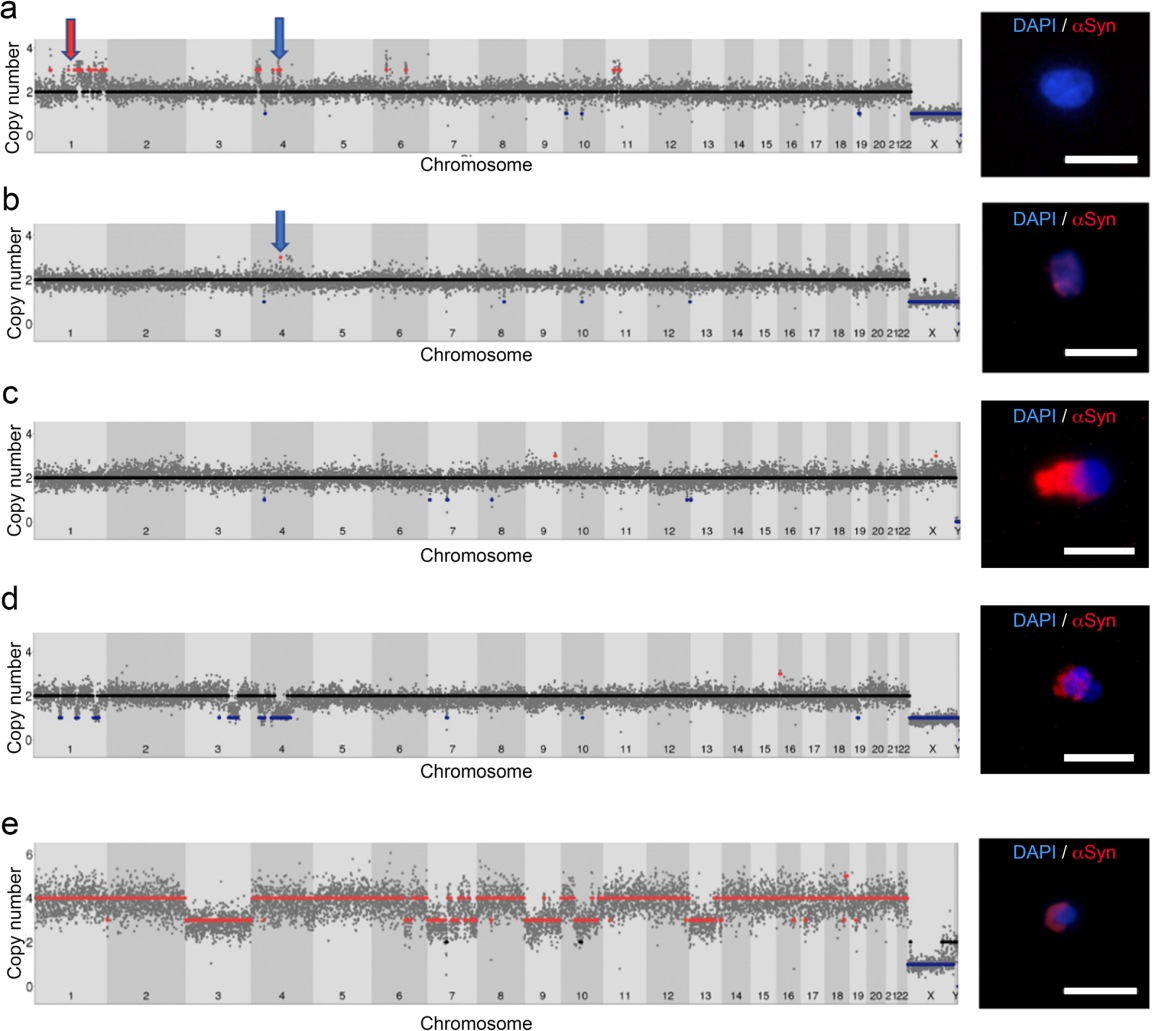
Examples of single cell WGS profiles showing CNVs. The WGS profile is shown for each, with a picture of the nucleus on the right. Scale bar 20 μm. Gains are losses are marked by dots at the respective copy numbers. The cell number is in brackets. **a** Pontine neuron with gains including *SNCA* (blue arrow), and adjacent to *GBA* (red arrow) (K3). **b** Pontine neuron with a nuclear inclusion and a gain over *GRID2* (arrowed) (X21). **c** Nigral neuron with a cytosolic inclusion and two gains (H11). The dots representing losses are CNVs that were filtered based on the copy number criterion, and therefore have not been included in the analysis. **d** Putaminal neuron with a nuclear inclusion and multiple losses, including the *SNCA* region (L33). See also Additional file 2: Figure S6d. **e** Pontine non-neuronal cell with a cytoplasmic inclusion and likely tetraploidy with superimposed losses (D8)

**Fig. 5:**
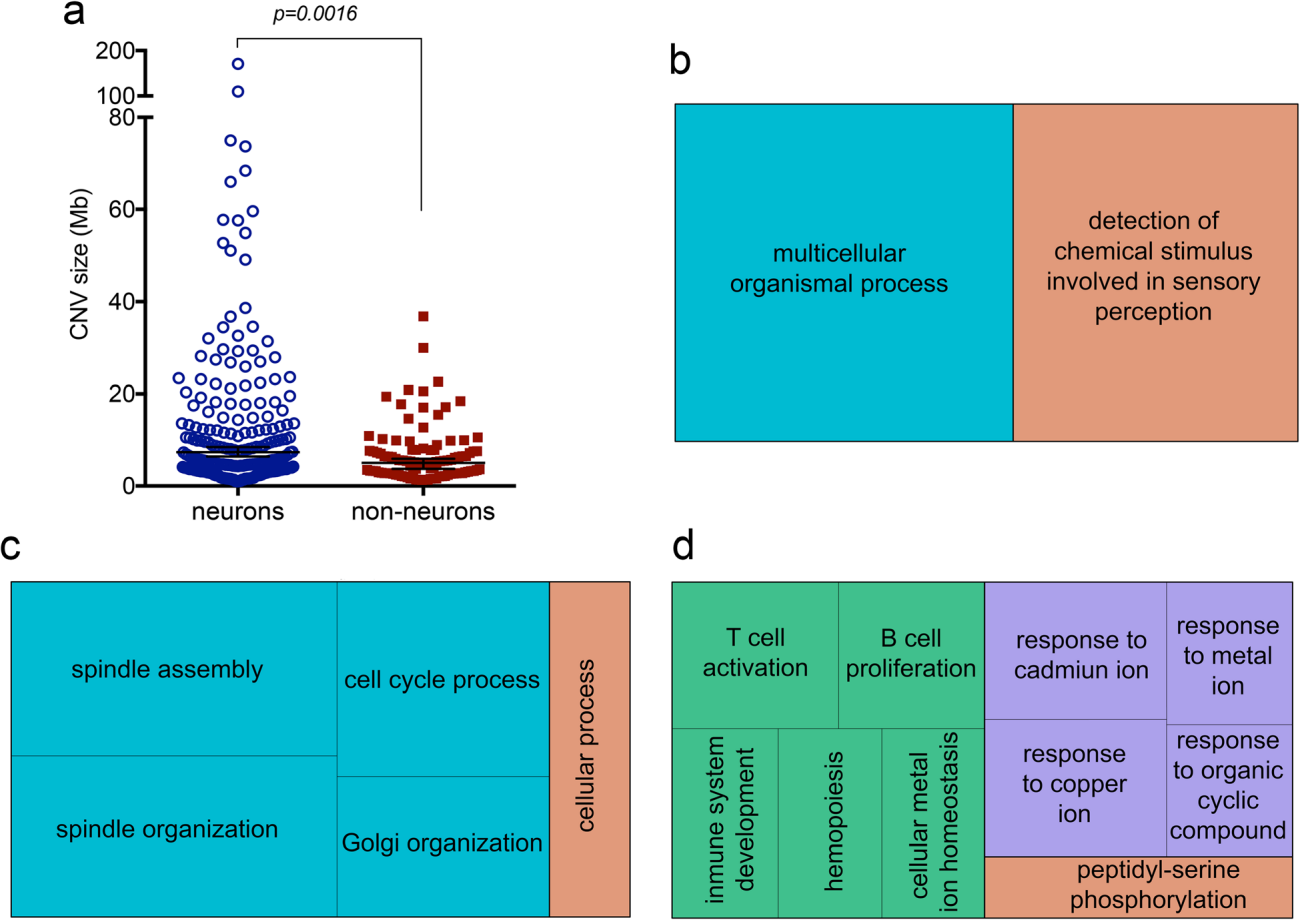
CNV size and pathway enrichment. **a** Comparison of size in Mb of sub-chromosomal CNVs in neurons and non-neuronal cells. **b**-**d** Gene Ontology maps showing biological processes enriched for CNVs in MSA in: (**b**) all neurons, (**c**) SN neurons, (**d**) SN non-neurons

We obtained 169 successful single cell CNV profiles from two MSA brains (Table 3). The overall success rate (proportion of sequenced cells passing QC) was 59.1%, or 65.6% excluding putamen, which performed markedly worse overall (35.5%; Fisher’s exact *p* = 0.0006). The highest success rate was in SN neurons (75.5%). We detected CNVs in 50 cells (29.6% of the total). These were found in all brain regions (nigra 30.3%, pons 31.2%, putamen 22.7%) (Fig. 4, discussed in detail later; Additional file 2: Figure S6). Cells which had CNVs frequently had more than one, and there were 313 CNVs in total (excluding polyploidy and “pure” aneusomies, where an entire chromosome was gained or lost with the same copy number throughout). These comprised 175 gains and 138 losses, and they were overall larger in neurons than non-neurons (medians 6.34 Mb v 5.05 Mb; Mann-Whitney *p* = 0.0016; Fig. 5a; Additional file 3: Table S6). Amongst cells with CNVs, the number of CNVs per cell was not significantly different in neurons and non-neurons (medians 2 and 1.5; Mann-Whitney *p* = 0.8195), or between cells with and without inclusions (medians 2 and 3 respectively; Mann-Whitney *p* = 0.6443). There were striking differences, however, between neurons and non-neurons in the *ratio of gains to losses*, with non-neurons having almost exclusively gains (95.1%), against 44.6% in neurons (Fisher’s exact *p* = 0.0001). Analysing the two SN specifically, gains predominated in both cell types, but more clearly in non-neurons (90.8%) than neurons (75.9%; Fisher’s exact *p* = 0.0427). Among olig2-positive cells, the only CNVs seen were gains, occurring in 50% (4/8) in the pons, but in none of the two in putamen. The presence of inclusions did not overall affect the type of CNV seen. Neurons with CNVs and inclusions comprised three with gains, two with losses, and two with both, while amongst those with CNVs but no inclusions, nine had gains, four had losses, and two had both. In non-neurons with CNVs, the ones with inclusions had gains in three, losses in one, and both in one, while in the ones without inclusions, 18 had gains, one had losses, and two had both.

**Table 3:** Summary of successful MSA single cell WGS

		Cells successfully sequenced					
		Neurons			Non-neurons		
Case	Region	Number	With inclusions	% CNVs	Number	With inclusions	% CNVs
MSA15 mixed	SN	22	1 C	40.9	30	3 C	23.3
	Pons	22	9 N, 2 C	27.3	26	7 C	30.8
	Putamen	13	6 N	23.1	9	3C	33.3
MSA10 SND	SN	18	2 C	33.3	29	10 C	30
Totals		75	15 N, 6 C	30.7	94	23 C	28.7

The number of cells of each type sequenced in each region / case is shown, together with the number which had inclusions (N = nuclear, C = cytoplasmic), and the % which had CNVs

We noted one pontine neuron with a 10.1 Mb gain straddling *SNCA* (Fig. 4a). This cell had multiple other CNVs, with clustered gains on chromosome 1q, and one reported breakpoint ~ 0.5 Mb from *GBA*. Several other examples of clustered gains were seen in the mixed MSA case (Additional file 2: Figure S6), including a pontine olig2-positive cell (K53) with extensive gains in four chromosomes, and a gain encompassing Parkin, which is somatically unstable in cancer [78]. A pontine neuron with a nuclear inclusion had a 1.36 Mb gain over *GRID2*, with a breakpoint ~ 2.6 Mb from *SNCA* (Fig. 4b). This very long gene is believed to be the key determinant of fragility in this region [100]. We detected small CNVs in all three SN neurons with inclusions (example in Fig. 4c), while only 29.7% of SN neurons without inclusions had CNVs (*p* = 0.037). This difference was driven by the SND case, where both neurons with inclusions, but only 2/16 without, had CNVs (*p* = 0.039). Losses occasionally predominated in neurons, such as a putaminal neuron with a nuclear inclusion and large losses on chromosome 4, including *SNCA* (Fig. 4d). Five clearly aneuploid or aneusomic cells, where at least one entire chromosome was gained or lost, were seen, with differing patterns in neurons and non-neurons, comparable to the differences seen for sub-chromosomal CNVs. Chromosome gains were seen in two non-neurons, including one, with a cytoplasmic inclusion, which had a profile consistent with a tetraploid cell which had lost some chromosomes (Fig. 4e). A similar profile has been reported before in human frontal cortex in a cell of unclear nature [51]. Three neurons had only losses, resembling control neurons already reported (e.g. Figure S3N in [70]). Interestingly two of these, in the mixed MSA pons, had further breakpoints with loss of most of the second copy of some affected chromosomes (cells X11, X16).

### Somatic CNVs can be clonal, and show enrichment for certain features

We reviewed all calls for evidence of possible clonality, indicated by CNVs with breakpoints within ~ 1 Mb of eachother, and found three such examples (Additional file 2: Figure S7a-d; Additional file 3: Table S6). In the mixed MSA SN, there was a ~ 3.5 Mb gain in two non-neurons (G13 and G72), although the apparent copy number was 3 in one and 4 in the other. A 4.85-Mb gain sharing the centromeric breakpoint was also seen in a pontine neuron from the same case (X14). Interestingly, the region within which the centromeric boundaries lie includes *MAPT*, and contains several segmental duplications (SDs) (Additional file 2: Figure S7a). In the SND MSA SN, two cells had a gain with one identical breakpoint, with respective sizes 1.9 and 3.2 Mb (H11, neuron with an inclusion; F30, non-neuron). *TLR4*, which is significantly upregulated in MSA SN and striatum [12], occurs between the reported centromeric boundaries, while the shared telomeric boundary is in a region with several SDs (Additional file 2: Figure S7b). In the pons, two non-neurons (K53, X14) had multiple gains in chromosome 5, and olig2 staining performed for one was positive. Two of these gains suggest possible clonality, as one CNV boundary differed by only one bin, while the other differed by ~ 3.5 and 1.7 Mb, and all boundaries were near SDs (Additional file 2: Figure S7c,d). These cells may therefore have shared lineage, with these CNVs established first, and additional ones through further mitoses. Alternatively, this region may be prone to recurrent events. To systematically investigate a possible relation of SDs, fragile sites, and telomeres with somatic CNVs, we looked for enrichment of these features at all CNV boundaries. We noted that 10.03% of CNV boundaries were in regions enriched for SDs, more frequently in neurons (10.49%) than non-neurons (9.05%; *p* = 0.0247). Four single cell CNVs had enrichment for SDs at both boundaries. Review of these revealed that three, including one from the cell with *SNCA* gain, had more than one pair of paralogous SDs very near the reported boundaries of each CNV, demonstrating a significant region of high sequence similarity across the two boundaries (Additional file 2: Figure S7e,f). We also noted that 6.7% of CNV boundaries were in telomeric regions, more in neurons (9.15%) than non-neurons (1.43%; *p* = 0.0006). We did not observe significant enrichment for fragile sites. We also wondered whether any of three genes reported in neuronal CNV hotspots [19] were involved in CNVs detected. We noted that one of them, *RBFOX1*, involved in neuronal splicing [114], was gained in three cells across both SNs.

Insights into any possible functional effects of CNVs can be gained by determining the types of genes affected. We therefore first performed gene ontology analysis of CNVs in all neurons and non-neurons from both cases. Neurons showed enrichment in “detection of chemical stimulus involved in sensory perception” and “multicellular organismal processes” (Fig. 5b). The former includes the most enriched term in a previous analysis of control cortical neurons (“sensory perception of smell”), and the latter includes most of the other categories reported [19]. There was no significant enrichment in non-neurons overall. We next analysed MSA SN neurons and non-neurons from both cases, and the single cortical neuron CNVs reported in non-diseased individuals in a previous study for comparison [70] (Additional file 3: Table S7). MSA SN neurons showed clear over-representation of mitosis-related categories (“spindle organisation” and “cell cycle process”) in the “biological processes” annotation module (Fig. 5c). This was also evident in each SN when analysed independently (Additional file 2: Figure S8; Additional file 3: Table S8). MSA SN neurons also showed over-representation of Golgi-related genes in both the “biological process” and “molecular function” annotations. There was no suggestion of an over-representation of either these categories in MSA SN non-neurons; there was, however, a trend towards over-representation in the control neuronal data set. These processes therefore may be more likely to be affected by CNVs in neurons overall. Non-neuronal cells from the SN showed over-representation in genes involved in response to copper and cadmium, and metal ions overall. Similar over-representation was also seen in control neurons, but not in MSA SN neurons. The other main categories over-represented in MSA SN non-neurons were lymphocyte-related, and peptidyl-serine phosphorylation (Fig. 5d). There was no suggestion of over-representation of these in either set of neurons. These changes therefore appear specific to non-neurons, but it remains to be determined which glial type they occur in. In the “protein class” module, the main finding of interest was the over-representation of the MHC genes in MSA SN neurons, but not in non-neurons, or control neurons. It is not clear at this stage whether the changes limited to SN neurons or non-neurons are specific to MSA, or due to the intrinsic properties of these cells in the SN.

## Discussion

The existence of a wide range of somatic mutations in the brain, leading to differing genomes between cells (mosaicism), has become clear in recent years [71, 99]. This appears relevant to disorders of neurodevelopment [23], including intractable epilepsy [105], and neurodegeneration [58, 83, 113]. We previously reported the first evidence of somatic CNVs (gains) of *SNCA*, the gene encoding α-synuclein, more common in PD SN than controls [79]. Mosaicism in DA neurons (detected by the presence of neuromelanin, NM+) was found universally in cases with symmetric onset and no tremor, but not always in cases with tremor and / or asymmetry [79]. We have now used our FISH method to detect and quantify *SNCA* CNVs (gains) in individual neurons and other cells in the cingulate cortex, and expanded on previous work in the SN. As we were analysing sections, where losses could result from sectioning artefacts, we did not attempt to call these, consistent with our previous work. We also obtained preliminary data suggesting the existence of this phenomenon in the putamen, occipital cortex, and pons.

In the region we mostly focused on, the cingulate cortex, we found higher neuronal *SNCA* mosaicism in disease than controls (2.8% in MSA, 2.3% in PD). In MSA there was also a significant difference in non-neurons, as perhaps expected if these CNVs are relevant in a disease with prominent non-neuronal involvement. The apparent association of higher cingulate neuronal mosaicism with younger age of death in PD requires validation in larger sample sizes, and should be studied in other regions. It could indicate a detrimental effect of higher neuronal mosaicism. In MSA SN, we demonstrated *SNCA* mosaicism levels > 3% in NM+ (dopaminergic) and NM- (non-pigmented) cells. In three MSA-SND analysed further, we confirmed that NM- cells with CNVs included cells positive for olig2, an oligodendroglial marker. Indeed, these had higher levels of mosaicism than NM−/olig2- cells. It will be interesting to investigate this in more cases, including additional characterisation of NM−/olig2- cells. In this exploratory study, we did not find any significant correlations of mosaicism with age of onset, age of death, disease duration, or severity of GCI pathology. There was a trend for earlier onset being associated with higher mosaicism in NM- cells in MSA-SND (*r* = − 0.6), but this was not significant (*p* = 0.074), unless a case with unusually late onset was removed (*r* = − 0.78, *p* = 0.018). Further investigation of this in more cases with onset ages in the typical range is clearly warranted.

A possible functional role for somatic *SNCA* CNVs in the SN is suggested at the single-cell level by their apparent association with the presence of α-synuclein inclusions in the same cell, in a cell-type specific manner: in NM- cells in MSA-SND (RR 4.16), and in NM+ cells in LB cases (RR 6.03). A plausible, though unproven, explanation is that *the SNCA gain contributes to the development of the inclusion in the same cell* through increased mRNA expression. We note, however, that the sample size used is small, we did not measure mRNA levels, and further work is thus needed to assess the biological significance of this preliminary finding. Furthermore, most cells with *SNCA* CNVs did *not* have detectable inclusions, for which there are multiple non-mutually exclusive possible explanations: (i) CNVs are not always functional; (ii) some cells can counter the effects of higher *SNCA* gene dosage; (iii) FISH pre-treatment, which includes pepsin, removed the more peripheral or less robust inclusions; (iv) these cells may contain smaller pathological conformers, such as oligomers, detected using proximity ligation assay in PD [97] and MSA [102]. Conversely, most cells with inclusions did not have detectable CNVs, which could be due to: (i) false negatives due to overlapping FISH signals; (ii) presence of other detrimental CNVs; (iii) increased *SNCA* expression due to epigenetic modifications; (iv) a different cause of the inclusion, including a toxic cellular environment or spread of α-synuclein.

We believe our current and previous data suggest a *possible role for somatic SNCA CNVs in the aetiology and pathogenesis of synucleinopathies*. An origin in early embryogenesis is possible, as human and mouse neurogenesis leads to neuronal somatic mutations [5, 98], with CNVs specifically demonstrated to arise during mouse neurogenesis [98], and the presence of fewer neurons with CNVs in aged control human brains is consistent with an early origin [19]. The apparent paradox of developmental CNVs contributing to pathology which arises much later in life is best explained by the analogy with cases of inherited (germline) mutations: carriers of *SNCA* CNVs, missense mutations, or indeed other relevant mutations, by definition have them in all cells from conception, yet they often appear normal until middle age. There is still debate about the origin of aggregated α-synuclein in MSA, but exogenous α-synuclein can act as the trigger for oligodendroglial aggregation in MSA models [45, 69]. Our data are consistent with this, with somatic CNVs in both cell types possibly having a role. A functional CNV in an oligodendrocyte can increase the risk of a GCI in that particular cell, in response to exogenous α-synuclein released by neighbouring neurons, as the endogenous level, normally very low, will be higher. In a similar way, functional CNVs in DA neurons would increase the amount, and subsequent release, of α-synuclein. This proposed mechanism should also be considered in the context of the proposed prion-like spread in synucleinopathies, as cells carrying somatic mutations could generate the “seed” from which spread occurs [91]. The development of Lewy bodies in grafted embryonic neurons in PD, which has been repeatedly reported [20, 53, 61, 62], would be unlikely to be explained by somatic mutations in these neurons, although we note that these were not seen in all patients [73]. Microglial activation may also play a role in the development of inclusions in grafted cells [73, 87], and further work is needed to clarify the importance of protein spread in synucleinopathies [2, 72], and the possible relevance of somatic mutations to this.

To go beyond *SNCA*, we performed single cell WGS, using a manual nuclear isolation method previously used for neurons [19, 118]. Although limited in throughput, this allows tracing CNV calls back to individual nuclear characteristics and markers, and to the presence of nuclear α-synuclein inclusions, with cytoplasmic inclusions also retained in some cases. We demonstrated a multitude of CNVs genome-wide in the two MSA cases studied, and confirmed the existence of somatic gains over and around *SNCA*, and a large loss involving it, in the largest and most varied study of single cell megabase-scale CNVs from a human neurological disorder to our knowledge. The overall fraction of neurons with CNVs (30.7%) is higher than the reported frequency in control cortex (~ 10–25%) [19]. We also identified CNVs in a very similar proportion (28.4%) of non-neuronal cells. The only data from non-neuronal brain cells we are aware of (NeuN-negative from control cortex) showed a lower proportion of non-neuronal than neuronal cells with CNVs from the same three brains (7.0% v 13.0%) [19]. Our data therefore appear to suggest a higher proportion of neurons and non-neurons with CNVs than reported controls, but we should emphasise that these are from different brain regions, and obtained by different labs. We detected CNVs ranging from ~ 1 Mb (the smallest size we can confidently detect), to amplification of the entire genome. We noted five cells with aneuploidy or aneusomies, consistent with neuronal single cell WGS studies, where it ranged from 0.7–4.4% [14, 51, 70, 110].

Although the frequencies of cells with CNVs were very similar in neurons and non-neurons, neuronal and non-neuronal CNVs differed in the size, the balance of gains and losses, as well as the pathways affected in the SN. There were similar numbers of gains and losses in neurons, while gains significantly predominated over losses in non-neurons, unlike previous work in controls, where only 26.7% of CNVs in non-neuronal cortical cells were gains [19] (M McConnell, personal communication). Analysis of the functional gene categories preferentially affected by CNVs showed differences, with distinct pathways affected in SN neurons and non-neurons. Neuronal CNVs had over-representation of genes involved in mitosis-related processes, while non-neuronal CNVs were overall enriched in genes involved in lymphocyte differentiation, and response to metals, notably copper and cadmium. We did not, however, determine the specific cell types carrying these CNVs, or indeed whether they are limited to the MSA SN, and some categories were shared with published control cortical neuron CNVs. Sequencing of large numbers of precisely typed cells from MSA and control SN would be required to determine the possible relevance of these findings. Notably, MSA SN non-neurons showed prominent over-representation of copper and cadmium response genes in CNVs. This was not seen in MSA SN neurons, although it was present in the analysis of published control neurons. Heavy metal toxicity has been implicated in synucleinopathies, with a possible interaction of α-synuclein and copper [2], while the product of another PD gene, *ATP13A2*, may regulate cation homeostasis, and modulate cadmium toxicity [94]. We are by definition unable to determine if such CNVs might have existed in MSA SN neurons which died early; this problem is a major challenge in all similar work if specific somatic mutations lead to preferential neuronal death [58].

The mechanism and timing of the development of the CNVs detected remains to be elucidated. The possible clonality of some CNVs, and the frequent occurrence of SDs at boundaries, strongly support a mitotic origin, at least for these. As neurons are post-mitotic, this could be in early neurodevelopment, consistent with the evidence that DNA breaks occur in neuronal stem cells [116], CNVs arise in mouse neurogenesis [98], SNVs arise in human neurogenesis [5], and the frequency of neuronal CNVs in higher in younger individuals [19]. Clonality was also seen for some neuronal CNV calls in other work [15, 50]. Enrichment for SDs at human brain somatic CNV boundaries was previously reported, but with enrichment only in one boundary [50]. We also identified CNVs enriched for SDs at both boundaries, including three with very high sequence similarity at the boundaries, arising from the presence of multiple pairs of paralogous SDs. SDs are associated with germline CNVs which arise through non-allelic homologous recombination (NAHR) [65, 107], particularly in the case of large CNVs [21], and somatic CNVs in cancer [111]. It appears very likely therefore that these CNVs arise by NAHR between those SDs. We also noted enrichment of CNVs at telomeres, consistent with previous work in human and mouse neurons [19, 50, 98]. It is worth noting that sub-telomeric regions are heavily enriched for SDs [4]. Detailed definition of CNV boundaries would be required to determine whether some CNVs extending to telomeric regions arise by SD-mediated NAHR.

An additional feature of note was the observation of several gains on the same chromosome in several cells, both neurons and non-neurons, and indeed the neuron with the *SNCA* gain had several others on the same chromosomes, and across the genome. This is therefore similar to a control cortical neuron which had a large gain across *SNCA*, with several other gains, including other regions of chromosome 4 (cell FTCX 225 in [70]). In some cases, CNV clustering resembled the “fragmented aneuploidies” reported in the developing mouse brain, which could be misinterpreted as aneuploidies in metaphase FISH [98]. Multiple structural variants in a chromosome can occur together in cancer, with shattering (chromothripsis) followed by repair (chromoanasynthesis) [49]. Clustered gains in the germline (or very early development) may arise through ﻿serial template switching during DNA replication [82], with microhomology and / or insertions at the breakpoints [68], supporting a replicative mechanism as the origin of these CNVs. Indeed, a window in very early development is believed to account for multiple de novo CNVs, preferentially gains [63]. It is possible that a similar window exists in early neurodevelopment, with CNVs arising at this stage in mouse brain [98]. One particularly intriguing finding was two pontine cells with two possibly clonal gains on one chromosome, and several other gains on the same chromosome and elsewhere, unique to each cell. One of these cells was olig2-positive (K53), although the other appeared to be a neuron (X14). This could indicate a very early developmental origin of the shared CNVs, with subsequent additional gains acquired independently, although the possibility that these cells, or their precursors, underwent chromothripsis and chromoanasynthesis independently cannot be excluded.

As we did not detect clonality for most of the CNVs detected by single cell WGS, and FISH does not allow the sizing of *SNCA* CNVs, we cannot exclude the possibility that CNVs in *SNCA* or genome-wide develop late, even post-mitotically. Post-mitotic CNVs, even if arising as a result of the disease process, could also contribute to disease progression. We note nominally significant correlations of mosaicism levels between different cell types: in MSA SN, between NM+ and NM- cells, and in PD cingulate, between neurons and non-neurons. If these preliminary findings were validated in larger sample sizes, they would be consistent with a shared developmental origin, although a shared response to the disease process could not be excluded. There is evidence for somatic SNVs [64] and tandem repeat further expansions [39] arising in post-mitotic neurons, but not for CNVs to our knowledge, with the exception of the recent report of somatic *APP* recombinants in Alzheimer’s [57]. DNA synthesis in post-mitotic neurons may be possible as a result of aberrant cell-cycle re-entry in Alzheimer’s disease, with tetraploidy claimed to precede neuronal cell death [120], and a similar report in PD [42]. Single cell DNA sequencing in Alzheimer’s did not, however, reveal significant aneuploidy levels which would be expected in such a scenario [110], and the apparently tetraploid cell in our data, and a similar one which narrowly failed QC (Additional file 2: Figure S6b), were not neurons. Furthermore, explaining the statistically significant co-occurrence of *SNCA* gains and inclusions in the same cells of the category most relevant to each disease would require specific *SNCA* DNA synthesis occurring in response to protein aggregation or cellular dysfunction. We are not aware of any evidence or mechanism for aberrant cell cycle re-entry leading to only certain loci being duplicated, and active DNA synthesis should result in a typical sequencing profile determined by DNA replication timing in single cell WGS data [26]. Neuronal deletions are perhaps easier to explain as a post-mitotic phenomenon secondary to DNA damage, arising through non-homologous end-joining, the error-prone mechanism by which neurons repair DNA double-strand breaks [67]. It is indeed tempting to speculate that this may apply to the pontine and putaminal neurons with nuclear inclusions and extensive losses, including in one case *SNCA*, as α-synuclein inclusions may lead to DNA damage [76], and double-strand DNA breaks may be an early feature in Alzheimer’s disease [103]. Further technical improvements will be required to estimate the frequency of *SNCA* losse*s* by FISH.

The detection of single cell CNVs in the brain has now been reported by several groups. We used the newest version of the Picoplex WGA kit, which has been repeatedly shown to be highly appropriate for this purpose [24, 36, 66, 84]. For analysis, we used a widely used pipeline for initial calling, which has been found independently to be reliable for breakpoint and copy number calling [29], together with two QC filters derived from separate groups, one for single cell WGS quality, and one for individual CNV quality. We confirmed detection of two different germline *SNCA* CNVs in single cells, and also noted patterns of apparent false positives in our main dataset and removed these. We believe we have taken all steps possible to reduce false positives, and false negatives > 1 Mb, although by definition we cannot orthogonally validate calls in a single cell whose genome has been amplified. The frequency of *SNCA* CNVs by FISH in our samples appears higher than by single cell WGS. One possible explanation is the size. Our 50 kb FISH probe would detect even modestly sized CNVs, but inherited *SNCA* CNVs range from ~ 0.14–11 Mb [10, 28], with breakpoints within a ~ 12 Mb region around *SNCA* reported as a fragile site [100], and somatic *SNCA* CNVs may thus be sub-megabase. We are unable to detect any CNVs < ~ 1 Mb with our current single cell WGS methods, although this has been reported in mouse neurodevelopment [98] and using a micro-fluidic platform [38]. Full correlation of inclusions with single-cell WGS, and precise spatial information, would require laser-capture microdissection, as reported in breast cancer [17], and deeper sequencing or significantly improved WGA and bioinformatic methods to detect smaller CNVs. We should also emphasize that, in addition to “simple” CNVs of varying sizes, the healthy and diseased brain may harbour a wide range of structural variants and large-scale somatic genomic alterations, with recent reports in Alzheimer’s disease of somatic recombinants in *APP* [57], and herpes viral insertions [95]. None of these would be detectable in our current data set, and neither would be somatic “point mutations” (SNVs), hundreds of which may exist in each neuron [5, 64], unless much higher sequencing coverage were used.

In conclusion, we propose that somatic *SNCA* CNVs may have a role in the aetiology and pathogenesis of sporadic synucleinopathies. Their presence in the SN in the cell type most prone to inclusions appears to be associated with an increased risk of inclusions in the same cell, which we have analysed in the first study to our knowledge combining the detection of a specific somatic mutation and the relevant inclusion in a human neurodegenerative disorder, although large sample sizes will be needed to verify or refute this. The multitude of other single cell CNVs detected in MSA merit further study as possible contributors to aetiology or disease progression, although we are not at present able to infer causation.

## Supplementary information

**Additional file 1: Table S1.** Summary of demograhics of cases used, and all experiments performed on each. These are iindicated by “x” in relevant cell, with the following exceptions: The % SNCA mosaicism is provided by cell type for the SN and Cingulate cortex. The region(s) from which single cells were sequenced are shown in the relevant cells. The GCI grade is shown in the relevant cells. Note that the single DLB case was reported with ILBD in SN experiments. Empty cells signify that data not available, or experiment not performed. PMI = post mortem interval. OC = occipital cortex, PUT = putamen. sc = single cell. MPS = mate pair sequencing. AD = Alzheimer’s disease.

**Additional file 2: Figure S1.** Summary of bioinformatic pipeline for single cell WGS. **Figure S2.** Number of cells counted and analysed in each case / category in the cingulate cortex (a,b) and SN (c,d). **Figure S3.** *SNCA* CNVs and α-synuclein nuclear inclusions in MSA pontine neurons. **Figure S4.** Mate-pair sequencing results of MSA SNand cerebellum. **Figure S5.** Visual isolation of nuclei on an inverted microscope. **Figure S6.** Profiles of cells with CNVs. **Figure S7.** Detailed visualisation of boundaries of gains with evidence of shared breakpoints suggesting clonality, and gains possibly arising at segmental duplications (SDs). **Figure S8.** Pathway analysis of neuronal CNVs in each SN separately.

**Additional file 3: Table S2.** Mosaicism % in MSA-SND and mixed MSA in the cingulate cortex and substantia nigra. **Table S3.** Detailedcorrelation analyses of cingulate cortex and substantia nigra mosaicism. **Table S4.** Correlation of GCI in the cingulate cortex and sub-cortical region with mosaicism in MSA. **Table S5.** *SNCA* mosaicism in occipital cortex, putamen and pons. **Table S6** All CNVs which passed filtering. **Table S7.** Relative over-representation of gene categories in CNVs. **Table S8.** Enrichment factor in neuronal CNVs in SN.

## Data Availability

MSA sequencing data supporting the conclusions of this article are available at the European Nucleotide Archive https://www.ebi.ac.uk/ena . Accession numbers: mate-pair and single cell WGS PRJEB35076, exomes ERS3926266–82. R scripts used for calculation of confidence score are platform-independent, and provided at github.com/Proukakis (confidencescore.R).
