# Supplementary material for: Investigation of somatic CNVs in brains of synucleinopathy cases using targeted SNCA analysis and single cell sequencing
Source: Acta Neuropathol Commun. 2019 Dec 23;7:219. doi: 10.1186/s40478-019-0873-5 (PMC6929293; doi:10.1186/s40478-019-0873-5)
Supplement: Supplementary file 2 — Additional file 2: Figure S1. Summary of bioinformatic pipeline for single cell WGS. Figure S2. Number of cells counted and analysed in each case / category in the cingulate cortex (a,b) and SN (c,d). Figure S3. SNCA CNVs and α-synuclein nuclear inclusions in MSA pontine neurons. Figure S4. Mate-pair sequencing results of MSA SNand cerebellum. Figure S5. Visual isolation of nuclei on an inverted microscope. Figure S6. Profiles of cells with CNVs. Figure S7. Detailed visualisation of boundaries of gains with evidence of shared breakpoints suggesting clonality, and gains possibly arising at segmental duplications (SDs). Figure S8. Pathway analysis of neuronal CNVs in each SN separately. [file 40478_2019_873_MOESM2_ESM.pdf]

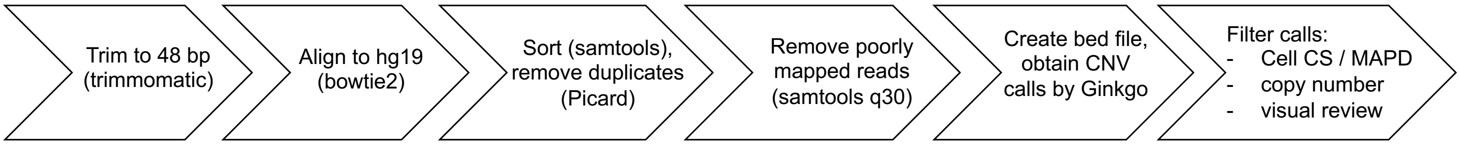

Supplementary Fig. S1. Summary of bioinformatic pipeline for single cell WGS.

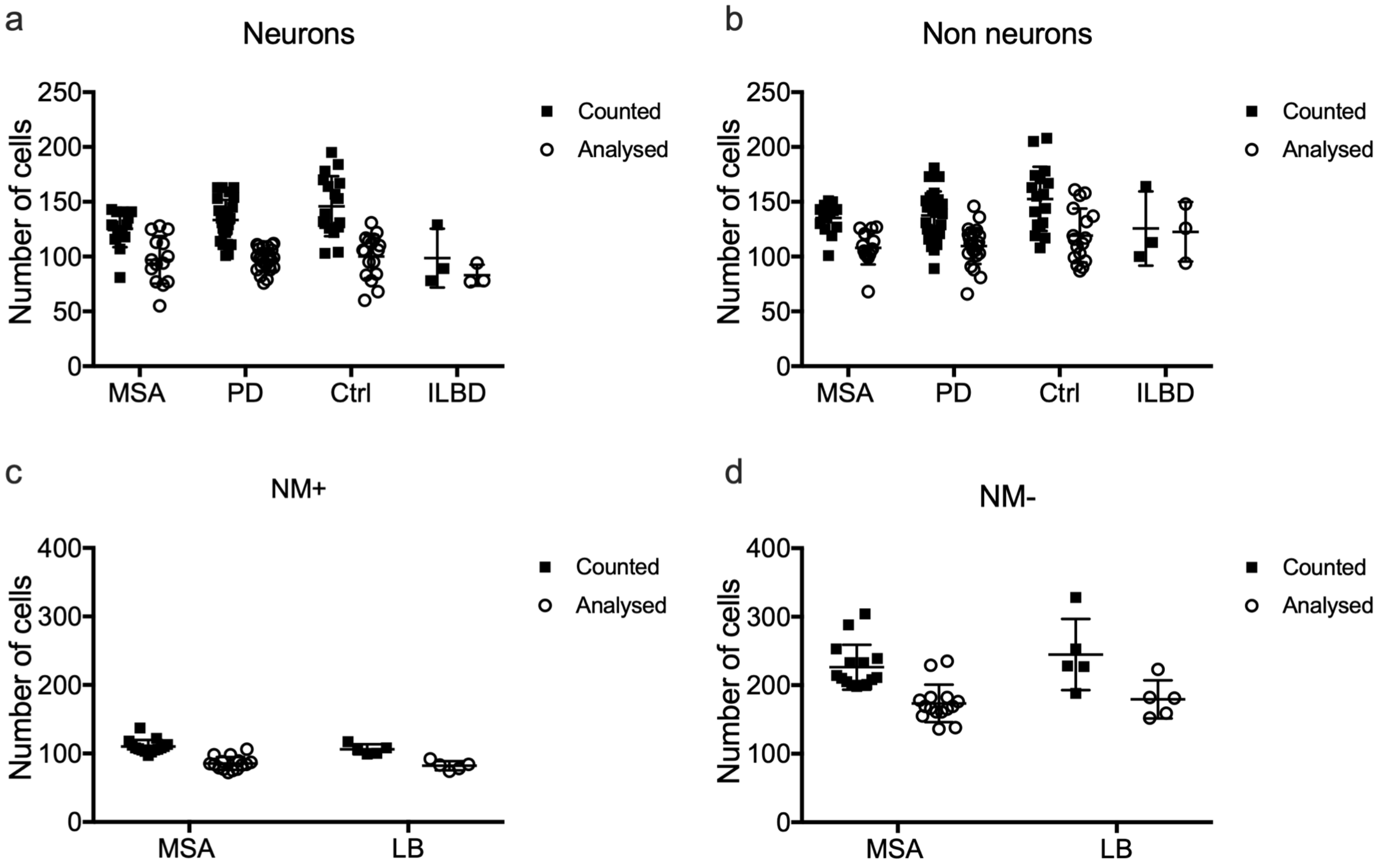

Supplementary Fig. S2. Number of cells counted and analysed in each case / category in the cingulate cortex (a,b) and SN (c,d). Mean and SD per category shown. The cell type is indicated (neuron v non-neuron, and NM+ v NM-). Note that there are two values for each group / case: on the left, all cells detected in photographs. On right, cells used for analysis: good quality cells with 2 copies of reference, and 2 or more copies of *SNCA* (see methods).

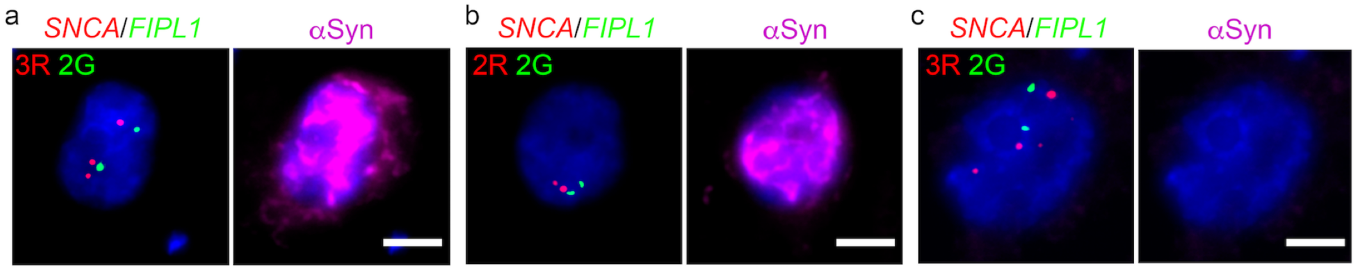

Supplementary Fig. S3. *SNCA* CNVs and α-synuclein nuclear inclusions in MSA pontine neurons.
A: a nucleus with inclusion and no CNV.
B: a nucleus with a CNV and an inclusion.
C: a nucleus with a CNV but no inclusion.
FISH for *SNCA*, reference. IHC for α-synuclein. Scale bars 5 µm.

Region	Fragment size (bp)	Bridged coverage	Base coverage	Unique fragments
SN	2,024	56x	2x	89.6%
Cerebellum	2,224	66x	2x	98.3%

High support breakpoints

1. Substantia nigra

RPs	Chr A	Chr B	Locus A	Locus B	Position A GRCh38	Position B GRCh38	Size	Gene A	Gene B	Position A GRCh37	Position B GRCh37
24	2	2	2q14.1	2q14.1	114,321,641	114,390,038	68,397			115,079,218	115,147,615
23	4	4	4p15.32	4p15.32	16,334,194	16,376,121	41,927			16,335,817	16,377,744
32	6	6	6q16.1	6q16.1	94,703,061	94,783,337	80,276			95,412,779	95,493,055
23	13	13	13q12.11	13q12.11	19,842,491	19,873,725	31,234	ZMYM5		20,416,631	20,447,865

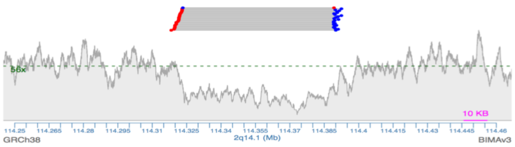

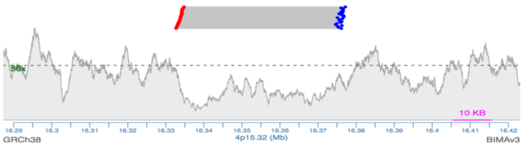

2. Cerebellum

RPs	Chr A	Chr B	Locus A	Locus B	Position A GRCh38	Position B GRCh38	Size	Gene A	Gene B	Position A GRCh37	Position B GRCh37
24	2	2	2q14.1	2q14.1	114,320,618	114,389,707	69,089			115,078,195	115,147,284
35	4	4	4p15.32	4p15.32	16,333,941	16,377,818	43,877			16,335,564	16,379,441
36	6	6	6q16.1	6q16.1	94,703,208	94,784,629	81,421			95,412,926	95,494,347

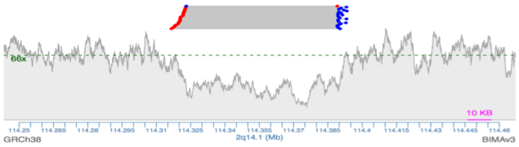

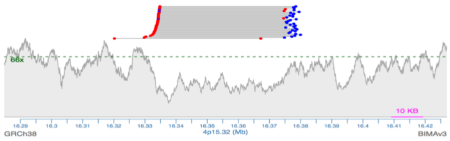

Supplementary figure S4 . Mate-pair sequencing results of MSA SNand cerebellum. This was performed as 100 PE in a single HiSeq2500 lane. The table shows the basic metrics for each. ”Base coverage” is the average coverage of all based, while “bridged coverage” is the average number of fragments spanning each base, which are interrogated for breakpoints. A list of high support breakpoints is provided for each sample. The coverage and spanning read pairs are shown for the first two deletions present in both samples, indicating a germline origin.

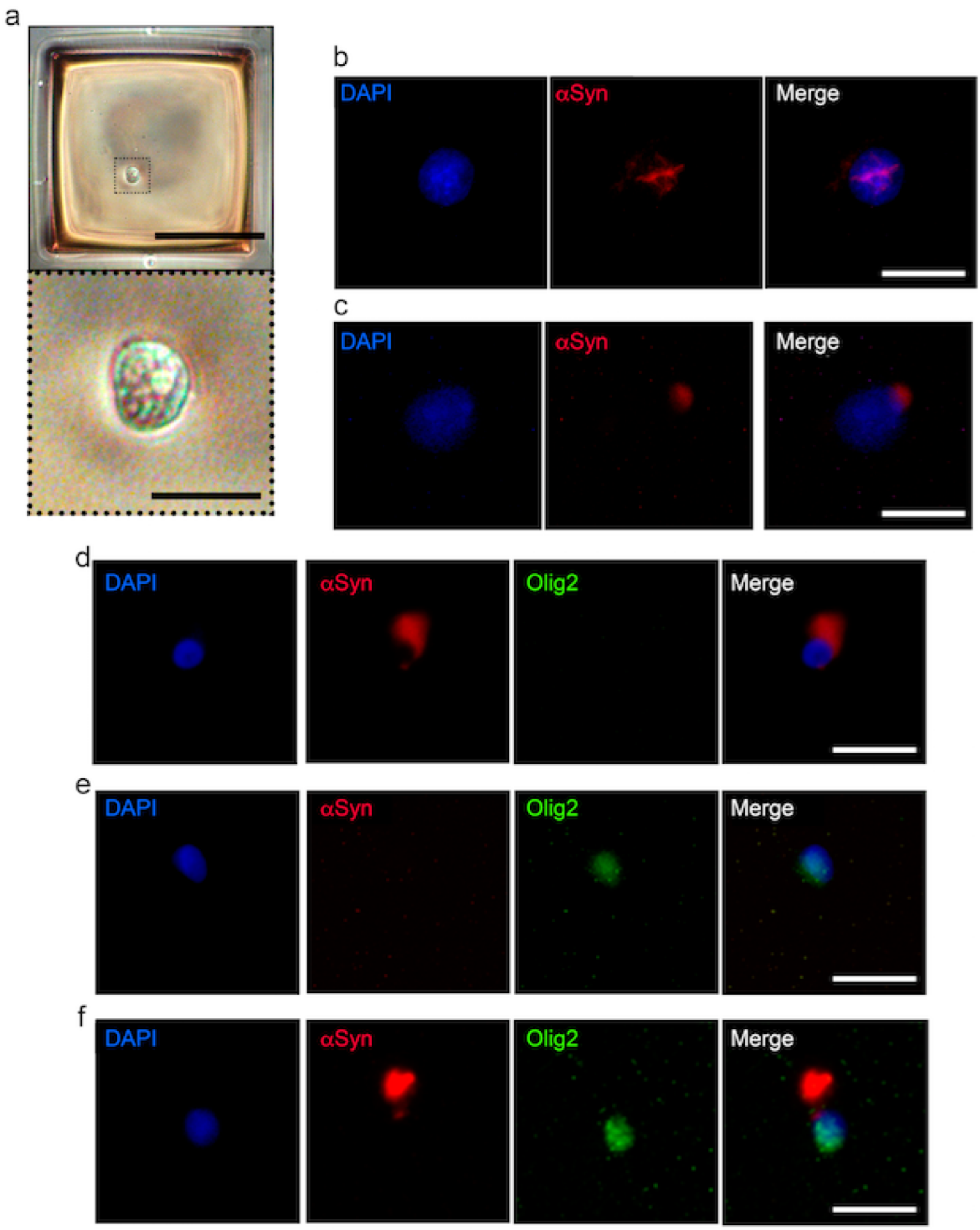

Supplementary **Fig. S5. Visual isolation of nuclei on an inverted microscope**.
a. Brightfield view of a raft from the CytoSort Array with a single nucleus, and enlarged version of dotted area below, which demonstrates the nucleolus. Scale bar 100 µm, inset scale bar 15 µm. **b**. Neuronal nucleus with nuclear αSyn inclusion. **c.** Neuronal nucleus with cytosolic αSyn inclusion**. d.** Non-neuronal Olig2- nucleus with a cytosolic inclusion. **e**. Olig2+ nucleus without inclusion. **f.** Olig2+ nucleus with a cytosolic inclusion. Scale bar b-f 20 15 µm.

Region	Cell	Type	Olig	Inc	CS	MAPD	reads	% Gen	PL	Sequencing profile
**SND MSA**										
SN	H11	1	0	2	0.80	0.19	1.93	0.26	2	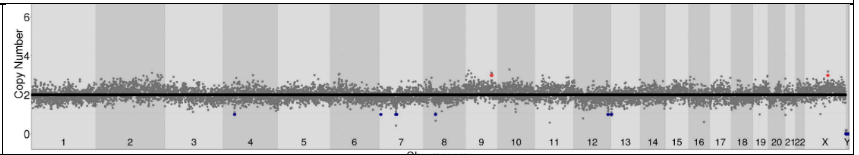
	F37	1		2	0.86	0.22	1.64	0.55	2	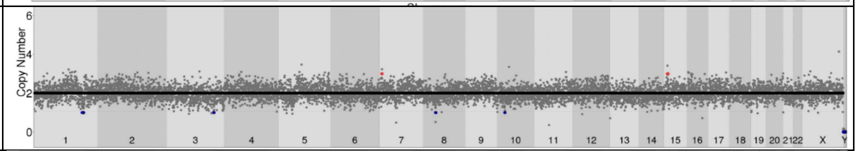
	F42	0		2	0.88	0.23	3.07	0.82	2	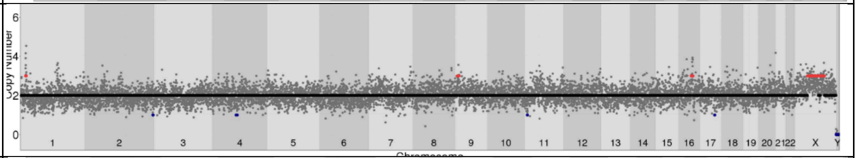
	F63	0		2	0.82	0.19	1.95	0.57	2	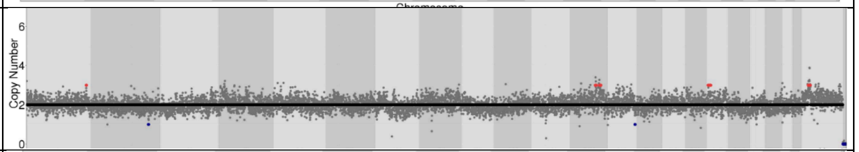
	F72	0		2	0.86	0.19	2.62	0.26	2	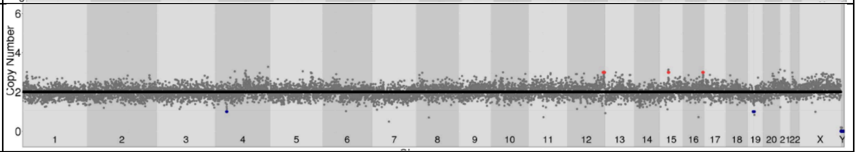
	F18	0		0	0.87	0.27	1.43	0.25	2	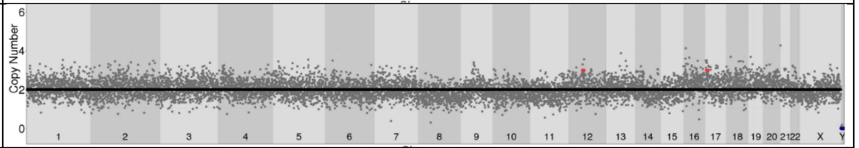
	F30	0		0	0.86	0.23	2.47	0.20	2	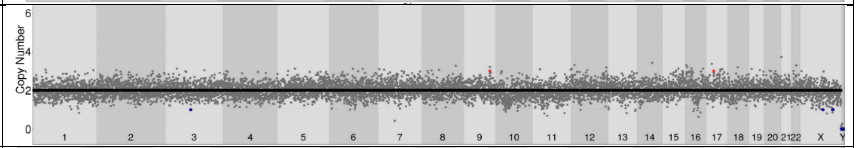
	F43	1		0	0.95	0.21	1.65	5.82	1.95	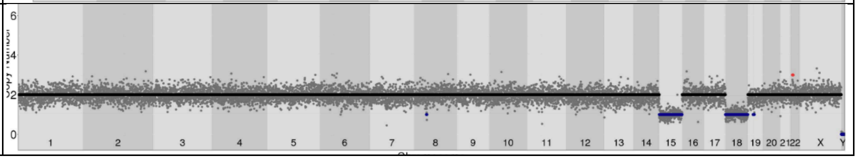

	F53	1		0	0.90	0.19	1.37	0.04	2	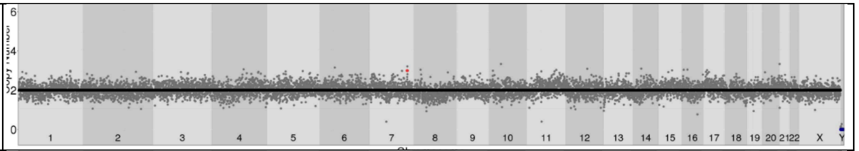
	F69	0		0	0.80	0.22	3.48	0.42	1.95	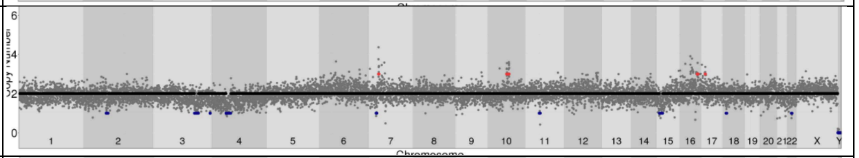
	F78	0		0	0.85	0.18	2.86	0.66	2	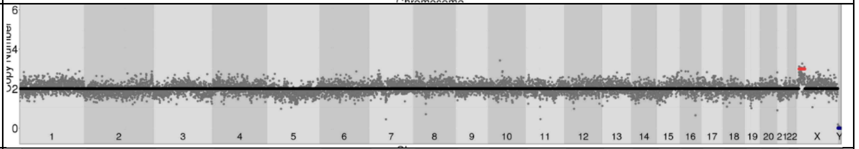
	F80	0		0	0.92	0.19	2.54	0.45	2	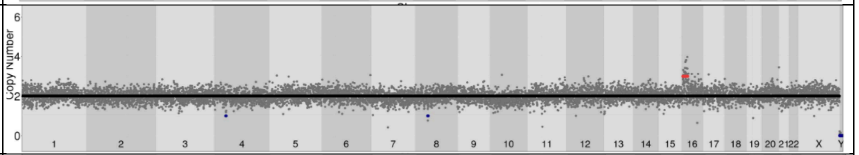
	F89	1		0	0.88	0.21	1.75	0.58	2	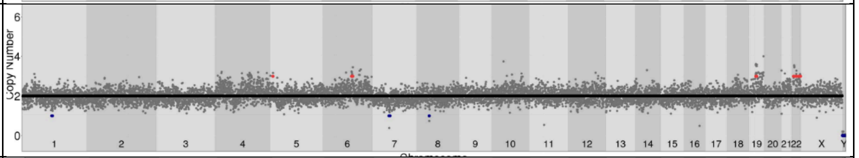
	H18	1		0	0.81	0.20	1.28	0.08	1.95	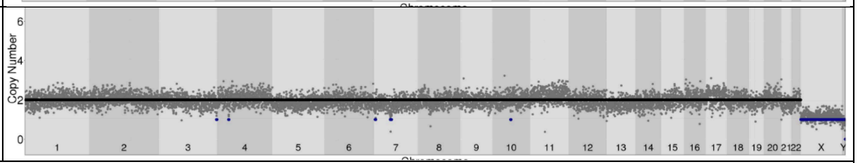
**Mixed MSA**										
nigra	H32	1		0	0.89	0.29	2.05	0.14	1.95	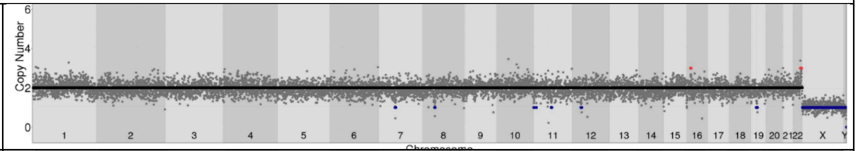
	G13	0		0	0.85	0.23	1.55	8.04	2.05	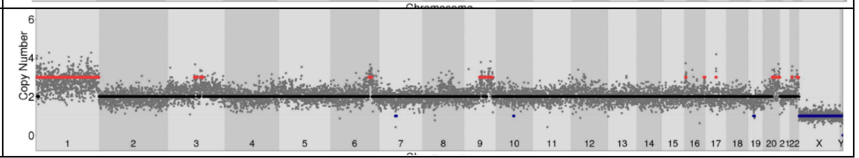

	G20	0		0	0.90	0.19	1.76	0.23	1.95	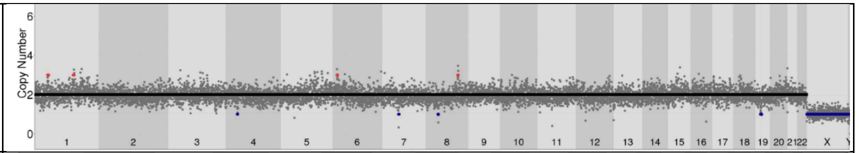
	G36	1		2	0.88	0.26	1.31	0.13	1.95	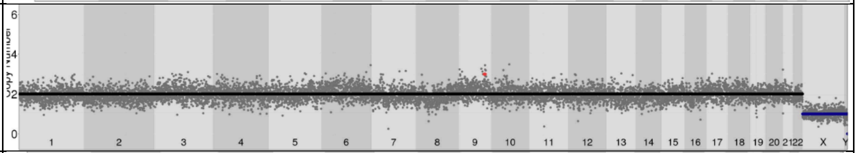
	G39	0		0	0.92	0.19	1.62	0.10	1.95	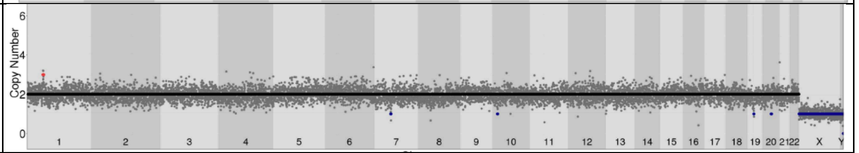
	G47	1		0	0.91	0.20	1.07	0.90	1.95	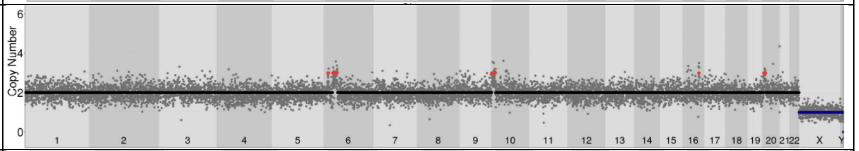
	G49	0		0	0.81	0.21	1.78	1.37	1.95	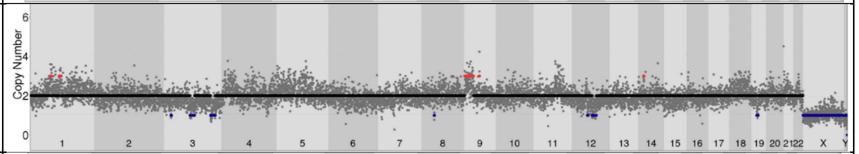
	G54	1		0	0.81	0.20	1.38	20.19	2.15	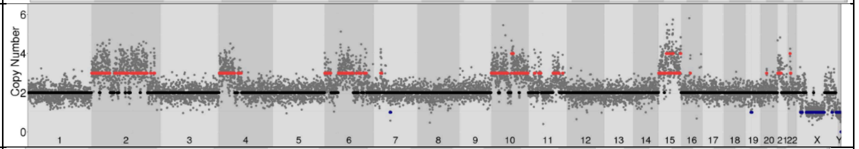
	G64	1		0	0.95	0.21	1.58	1.41	1.95	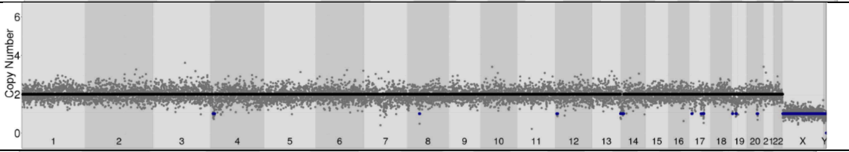
	G72	0		0	0.92	0.22	1.66	4.29	2.0	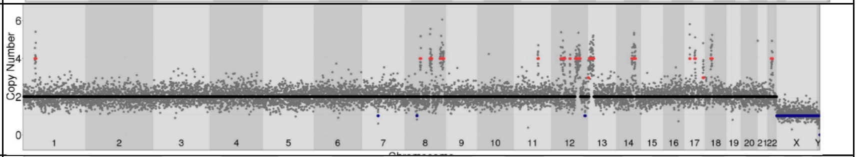
	G74	1		0	0.89	0.18	2.15	0.25	1.95	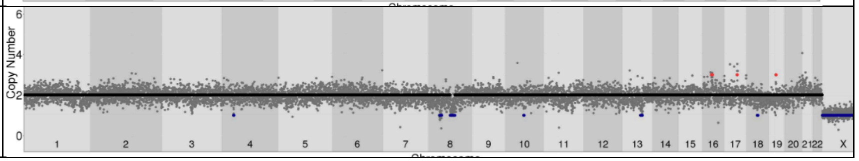

	G79	1		0	0.95	0.18	1.77	0.10	1.95	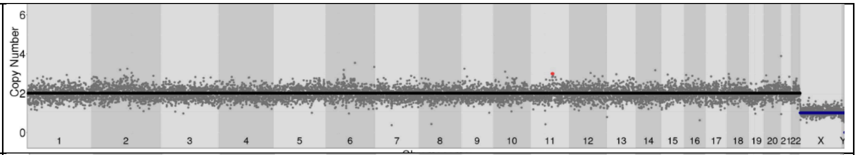
	G86	0		0	0.91	0.19	1.86	0.67	1.95	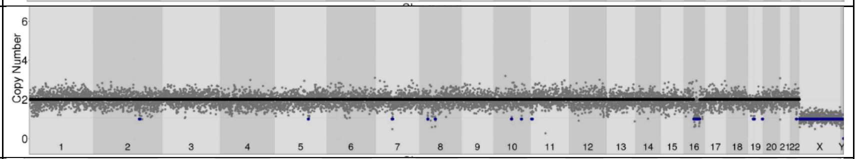
	G87	1		0	0.91	0.18	1.87	0.55	1.95	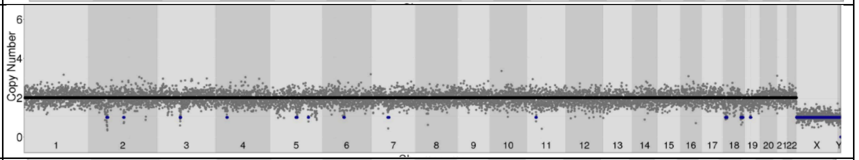
	G92	1		0	0.91	0.26	1.22	0.05	1.95	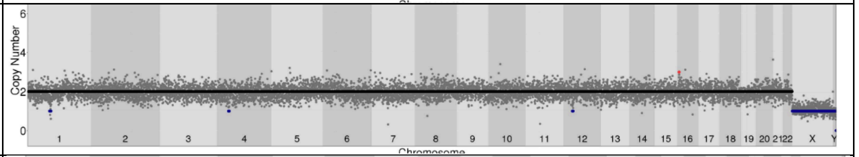
	H28	0	1	0	0.85	0.26	2.1	0.32	1.95	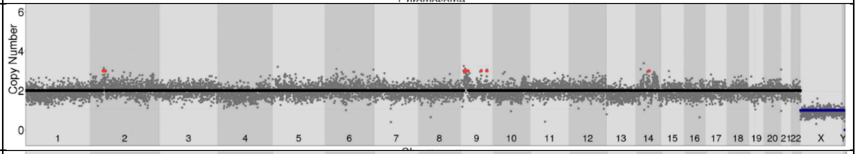
pons	X19	0		2	0.88	0.18	5.79*	0.73	1.95	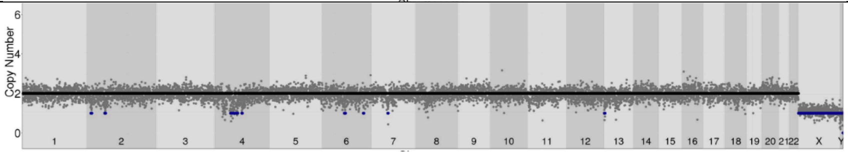
	D8	0		2	0.83	0.20	5.11*	96.76	3.6	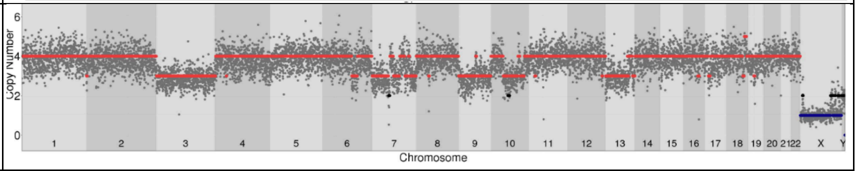
	X16	1		1	0.88	0.16	6.1*	27.92	1.6	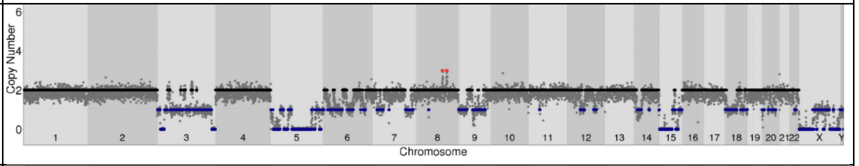
	X21	1		1	0.89	0.16	6.56*	0.09	1.95	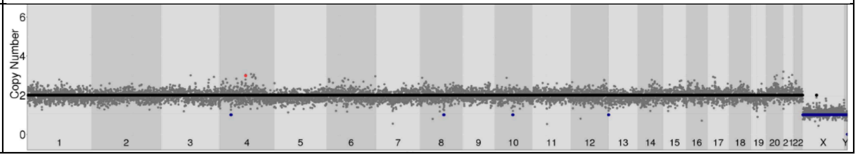

	D17	1		1	0.88	0.19	7.63*	0.22	1.95	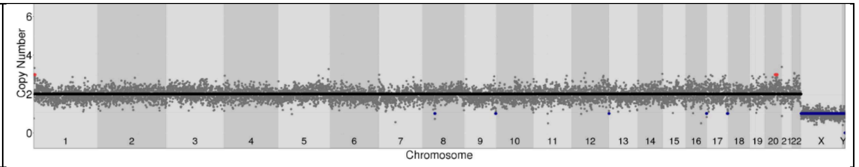
	K3	1	0	0	0.88	0.17	3.03	4.63	2.0	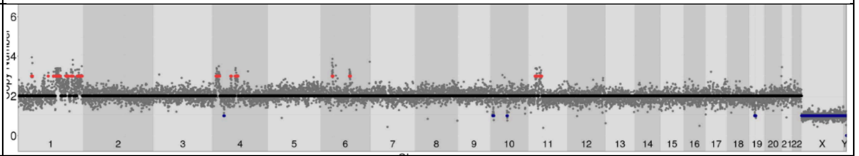
	K10	0	0	0	0.96	0.18	1.67	0.04	1.95	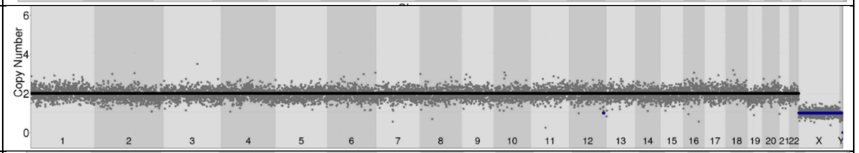
	K26	0	1	0	0.88	0.18	1.39	0.05	1.95	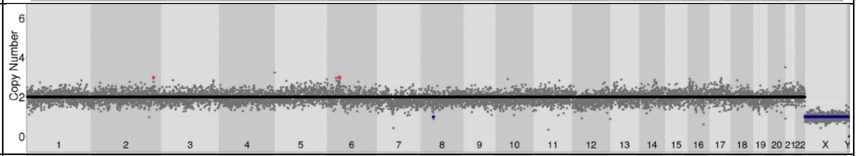
	K27	0	0	0	0.88	0.19	2.48	1.45	1.95	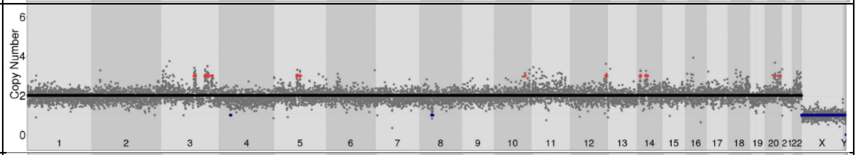
	K31	0	1	0	0.93	0.21	1.81	0.04	1.95	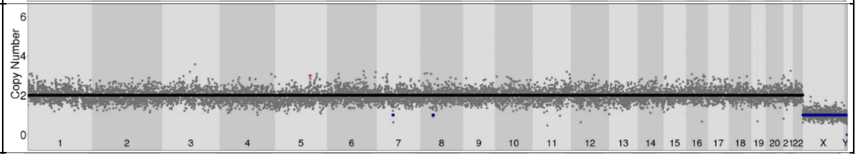
	K43	0	0	0	0.85	0.18	2.18	0.09	1.9	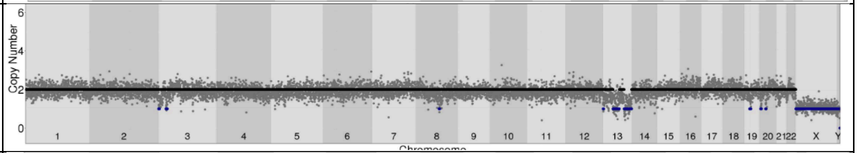
	K53	0	1	0	0.92	0.18	1.79	6.95	2.0	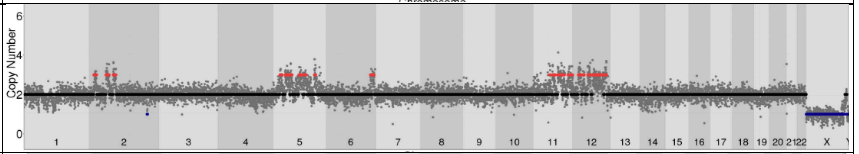
	K59	0	0	0	0.86	0.18	1.57	3.28	1.9	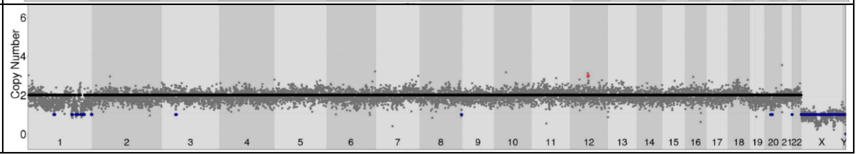

	X11	1		0	0.91	0.16	4.24	24.72	1.55	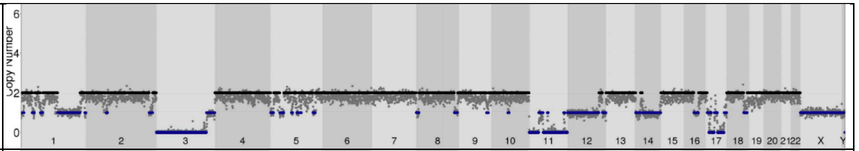
	X14	1		0	0.94	0.15	6.56	3.12	2.0	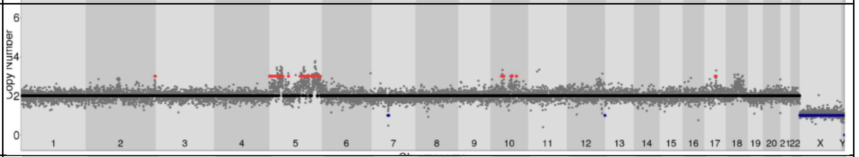
Putamen	L33	1	0	1	0.81	0.17	1.74	4.32	1.9	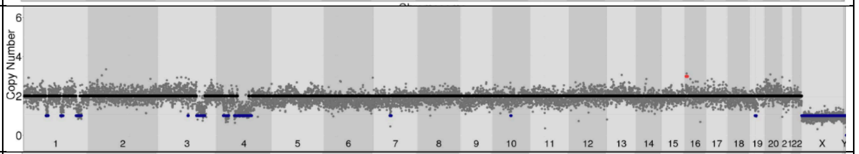
	L51	1	0	1	0.84	0.18	1.1	0.05	1.95	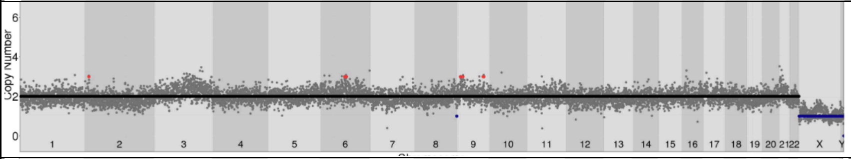
	L62	0	0	0	0.95	0.13	2.31	0.69	1.95	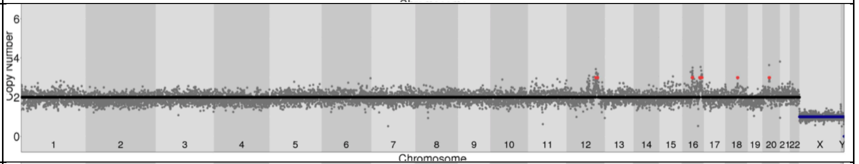
	L78	1	0	0	0.95	0.14	1.23	0.05	1.95	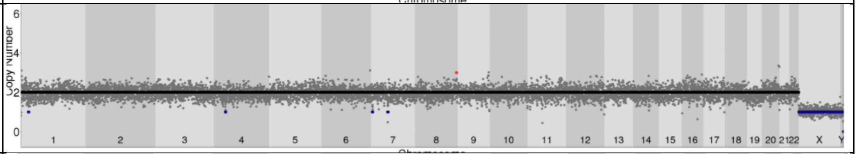
	L96	0	0	0	0.84	0.17	2.34	0.05	1.95	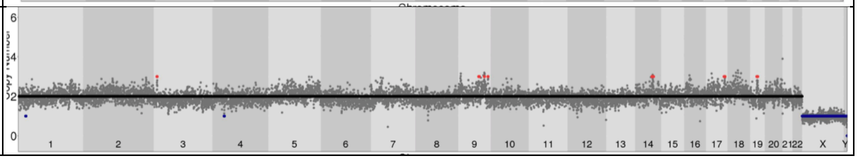

(b)

Region	Cell	Type	Olig	Inc	CS	MAPD	Reads	PL	Sequencing profile
Mixed MSA Pons	K39	0	1	0	0.798	0.16	2.32	4.1	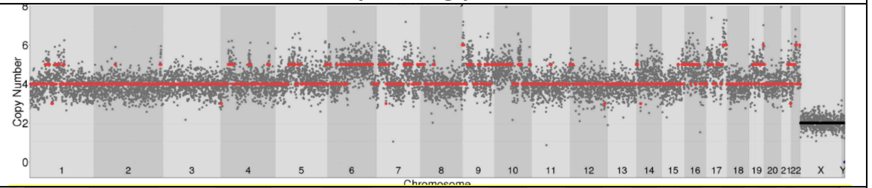
	K21	1	0	0	0.804	0.18	1.54	1.95	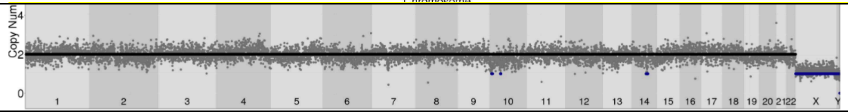
	K4	0	0	0	0.805	0.18	1.19	1.95	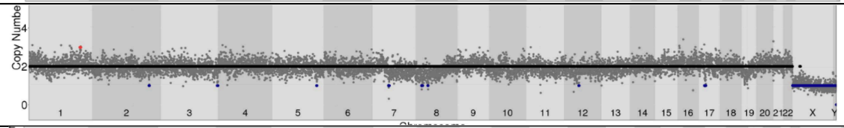
SND MSA Nigra	F76	0		2	0.827	0.17	2.31	2	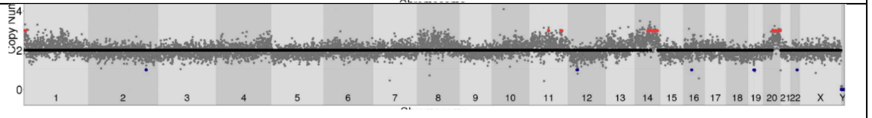

(c)

Region	Cell	Type	Olig	Inc	CS	MAPD	Reads	PL	Sequencing profile
SND MSA nigra	F50	0		0	0.94	0.19	1.33	2	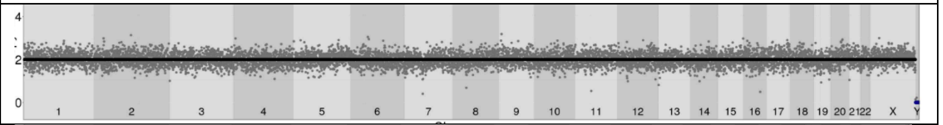
	F84	0		2	0.83	0.19	2.57	2	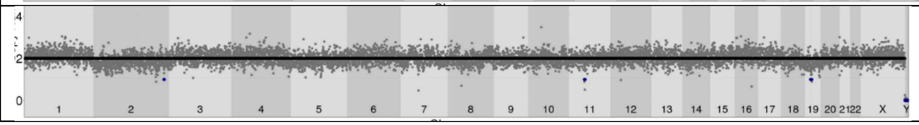

(d)

Region	Cell	PL	Sequencing profile at 1 Mb bin size
Mixed MSA putamen	L33	1.9	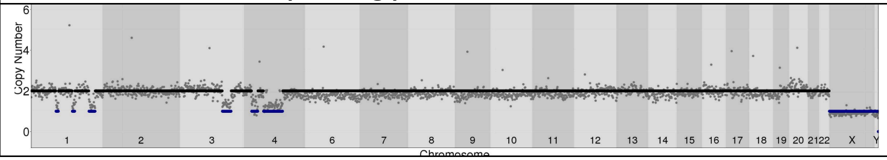

Supp fig 6: Profiles of cells with CNVs.

(a) All cells with CNVs which passed QC. “Type” 1= neuron, 2= non-neuron. “Olig” refers to olig2 staining; 1 if present, 0 if absent, blank if not performed. “Inc” refers to inclusion, 1= nuclear, 2= cytoplasmic, 0= none detected. CS= confidence score. PL= mean ploidy calculated by Ginkgo. “Reads”, denotes the number of reads; * denoted read pairs, as these cells underwent paired-end sequencing. “% Gen” is the % of the genome affected by CNVs. The co-ordinates of all CNVs are in Supplementary table S12. CNVs are indicated by dots / lines at the relevant copy number (red for gains, blue for losses). Note these symbols include CNVs called by Ginkgo which did not pass QC based on copy number. One loss (chr13 loss in K43) narrowly passed QC (1.44) but was in a very noisy region, with the whole chromosome slightly below copy number 2, and was rejected visually.

(b) Cells with CNVs which were narrowly rejected, and are therefore classed as cells with no CNVs.
- K39, a pontine early oligodendrocyte, was rejected based on CS. It appears tetraploid, similar to D8, another non-neuronal cell from the pons, for which olig2 staining was not available, but with superimposed gains rather than losses.
- K21, a pontine neuron which narrowly passed CS criterion. One of the chr10 losses passed the CN criterion (1.28) but was rejected as the entire chromosome appears below the baseline.
- K4, a pontine non-neuron which narrowly passed CS criterion. Two losses were rejected. The chr5 narrowly passed CN (1.43), but is a small (1.36 Mb) loss in a region where the chromosome baseline “dips”. One of the chr8 losses passed (CN 1.28), but the whole chromosome is below the baseline.
- F76, a nigral non-neuron with an inclusion. Although the cell passes CS, several chromosomes are noisy, with a wavy baseline. Calls which passed the CN filter were gains in chr1 (2.72) and chr14 (2.58), but were rejected as in a “wavy” area.

(c) Two normal cells shown for comparison, at the higher and lower end of quality as indicated by CS. F84 has 3 sub-threshold CNVs. The chr19 centromeric loss was frequently seen and rejected.

(d) The cell shown in Fig. 4d is shown here, with analysis at 1 Mb bin size, as this is a clearer visual representation of the losses.

Supplementary figure S7 (legend at end)

(a)

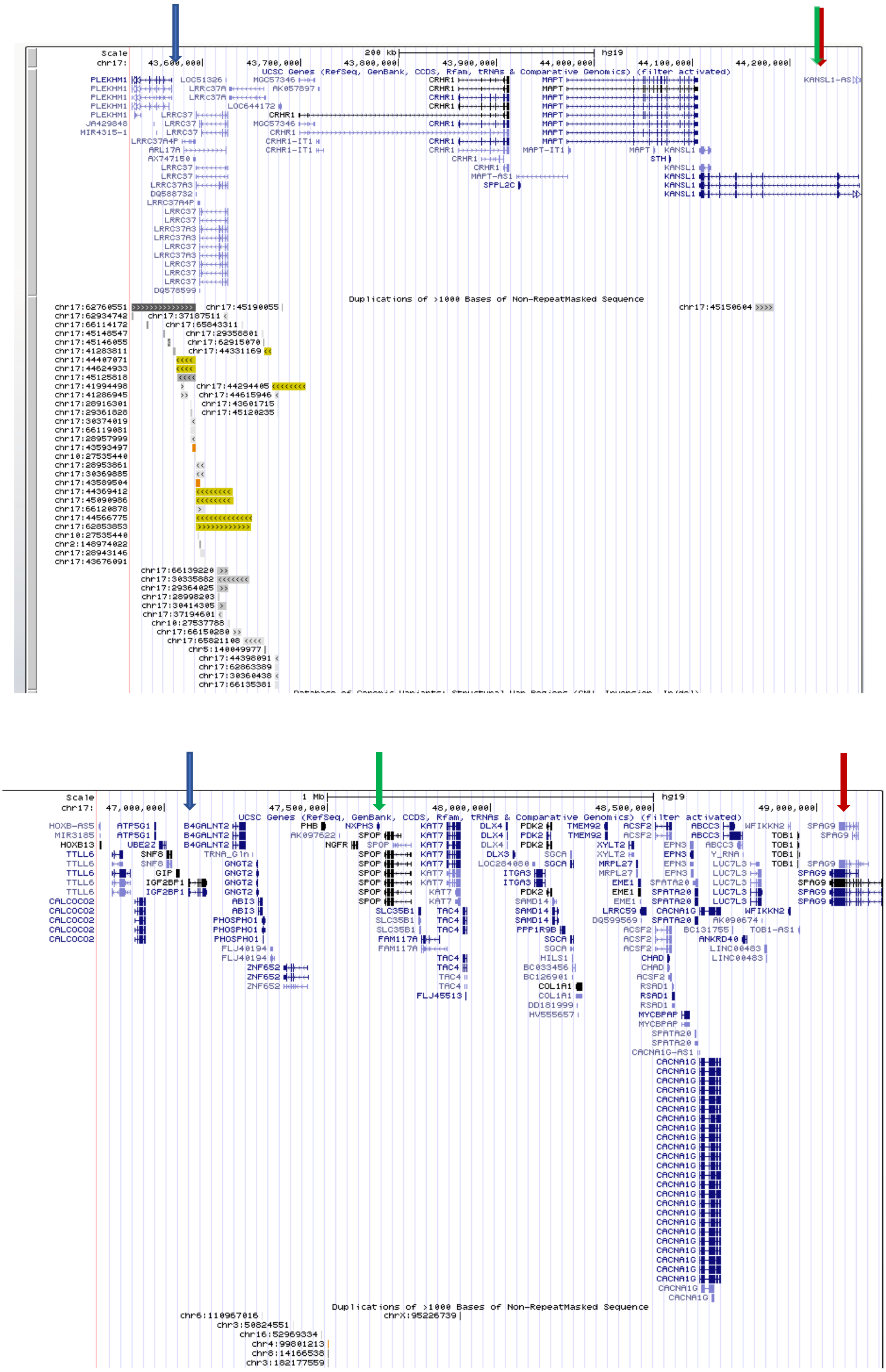

(b)

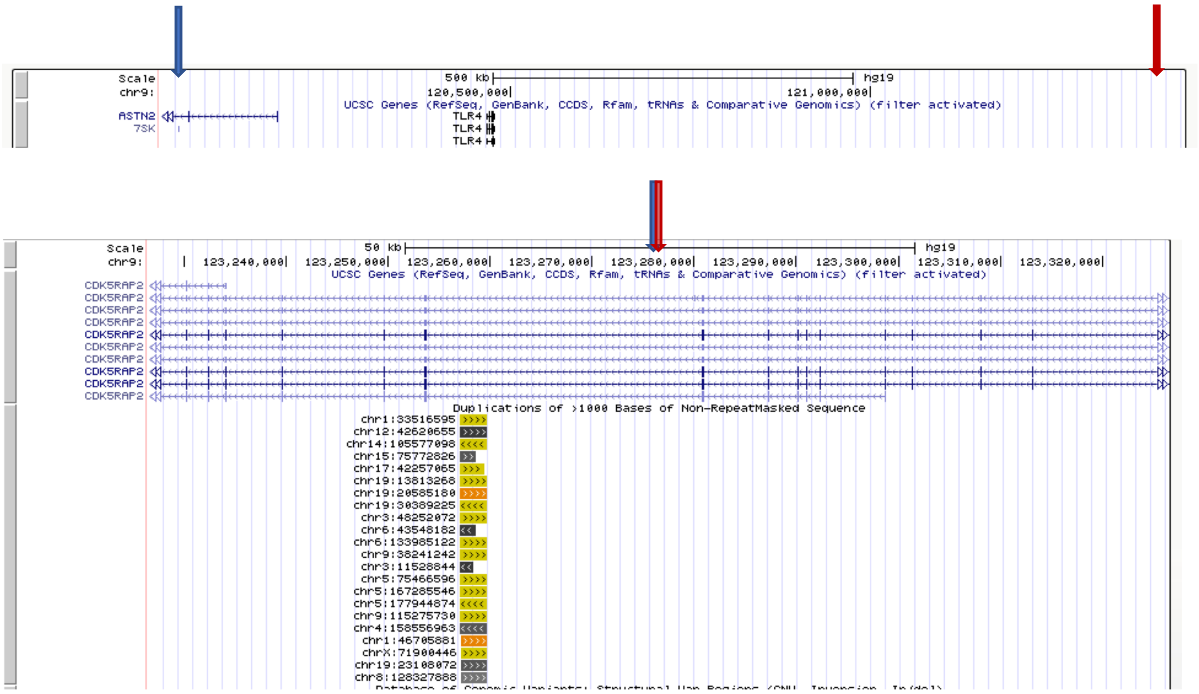

Scale
chr9:
500 kb
hg19
120,500,000
121,000,000
UCSC Genes (RefSeq, GenBank, CCDS, Rfam, tRNAs & Comparative Genomics) (filter activated)
ASTN2
7SK
TLR4
TLR4
TLR4
Scale
chr9:
50 kb
hg19
123,240,000
123,250,000
123,260,000
123,270,000
123,280,000
123,290,000
123,300,000
123,310,000
123,320,000
UCSC Genes (RefSeq, GenBank, CCDS, Rfam, tRNAs & Comparative Genomics) (filter activated)
CDK5RAP2
Duplications of >1000 Bases of Non-RepeatMasked Sequence
chr1:33516595
chr12:42620655
chr14:105577098
chr15:75772826
chr17:42257065
chr19:13813268
chr19:20585180
chr19:30389225
chr3:48252072
chr6:43548182
chr6:133985122
chr9:38241242
chr3:11528844
chr5:75466596
chr5:167285546
chr5:177944874
chr9:115275730
chr4:158556963
chr1:46705881
chrX:71900446
chr19:23108072
chr8:128327888

(c)

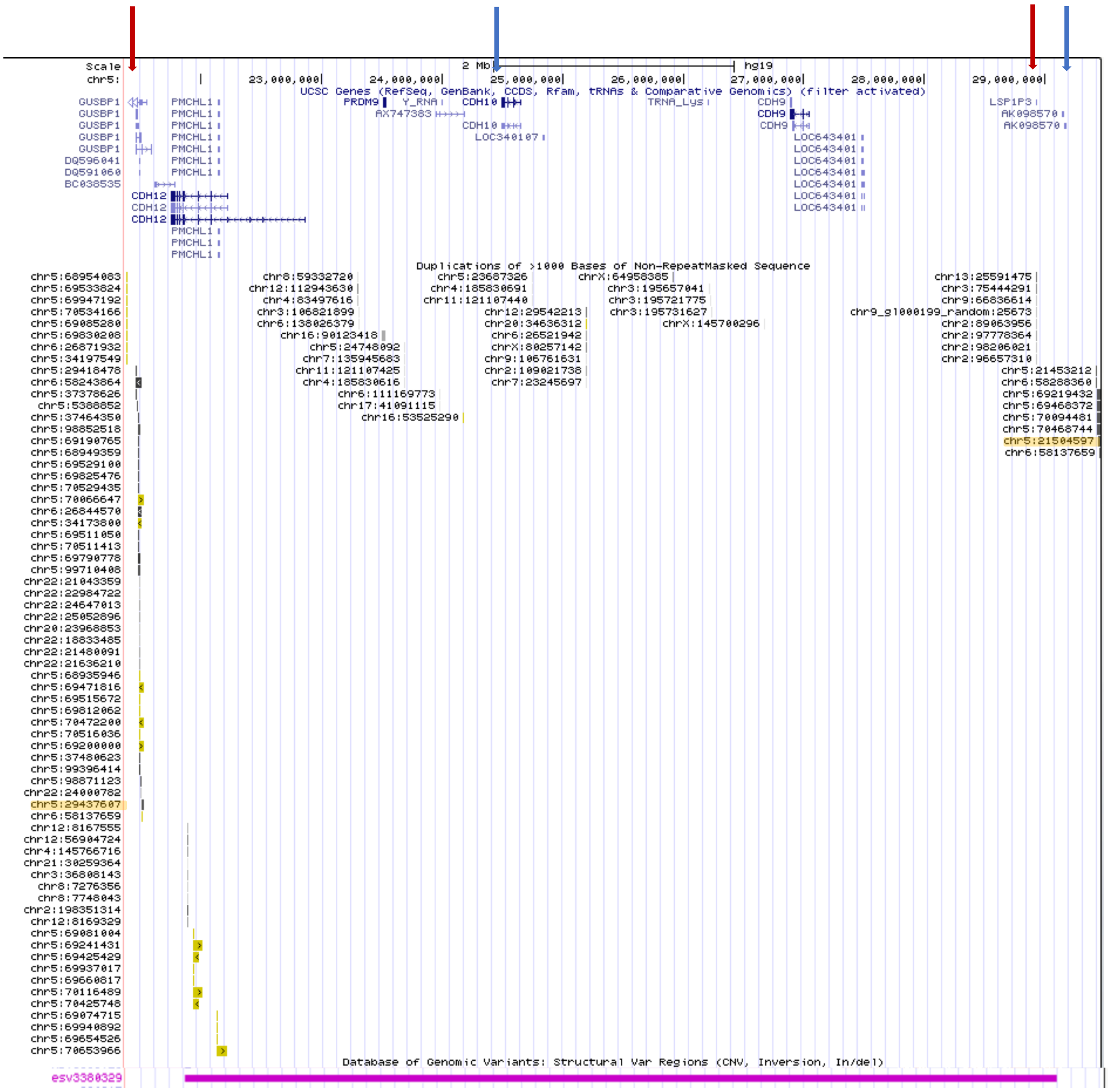

Scale
chr5:
2 Mb
hg19
23,000,000
24,000,000
25,000,000
26,000,000
27,000,000
28,000,000
29,000,000
UCSC Genes (RefSeq, GenBank, CCDS, Rfam, tRNAs & Comparative Genomics) (filter activated)
GUSBP1
PMCHL1
PRDM9
Y_RNA
CDH10
AX747383
CDH10
LOC340107
TRNA_Lys
CDH9
CDH9
CDH9
LOC643401
LSP1P3
AK098570
AK098570
DQ596041
DQ591060
BC038535
CDH12
CDH12
CDH12
Duplications of >1000 Bases of Non-RepeatMasked Sequence
chr5:68954083
chr5:69533824
chr5:69947192
chr5:70534166
chr5:69085280
chr5:69830208
chr6:26871932
chr5:34197549
chr5:29418478
chr6:58243864
chr5:37378626
chr5:5388852
chr5:37464350
chr5:98852518
chr5:69190765
chr5:68949359
chr5:69529100
chr5:69825476
chr5:70529435
chr5:70066647
chr6:26844570
chr5:34173800
chr5:69511050
chr5:70511413
chr5:69790778
chr5:99710408
chr22:21043359
chr22:22984722
chr22:24647013
chr22:25052896
chr20:23968853
chr22:18833485
chr22:21480091
chr22:21636210
chr5:68935946
chr5:69471816
chr5:69515672
chr5:69812062
chr5:70472200
chr5:70516036
chr5:69200000
chr5:37480623
chr5:99396414
chr5:98871123
chr22:24000782
chr5:29437607
chr6:58137659
chr12:8167555
chr12:56904724
chr4:145766716
chr21:30259364
chr3:36808143
chr8:7276356
chr8:7748043
chr2:198351314
chr12:8169329
chr5:69081004
chr5:69241431
chr5:69425429
chr5:69937017
chr5:69660817
chr5:70116489
chr5:70425748
chr5:69074715
chr5:69940892
chr5:69654526
chr5:70653966
chr8:59332720
chr12:112943630
chr4:83497616
chr3:106821899
chr6:138026379
chr16:90123418
chr5:24748092
chr7:135945683
chr11:121107425
chr4:185830616
chr6:111169773
chr17:41091115
chr16:53525290
chr5:23687326
chr4:185830691
chr11:121107440
chr12:29542213
chr20:34636312
chr6:26521942
chrX:80257142
chr9:106761631
chr2:109021738
chr7:23245697
chrX:64958385
chr3:195657041
chr3:195721775
chr3:195731627
chrX:145700296
chr13:25591475
chr3:75444291
chr9:66836614
chr9_gl000199_random:25673
chr2:89063956
chr2:97778364
chr2:98206021
chr2:96657310
chr5:21453212
chr6:58288360
chr5:69219432
chr5:69468372
chr5:70094481
chr5:70468744
chr5:21504597
chr6:58137659
Database of Genomic Variants: Structural Var Regions (CNV, Inversion, In/del)
esv3380329

(d)

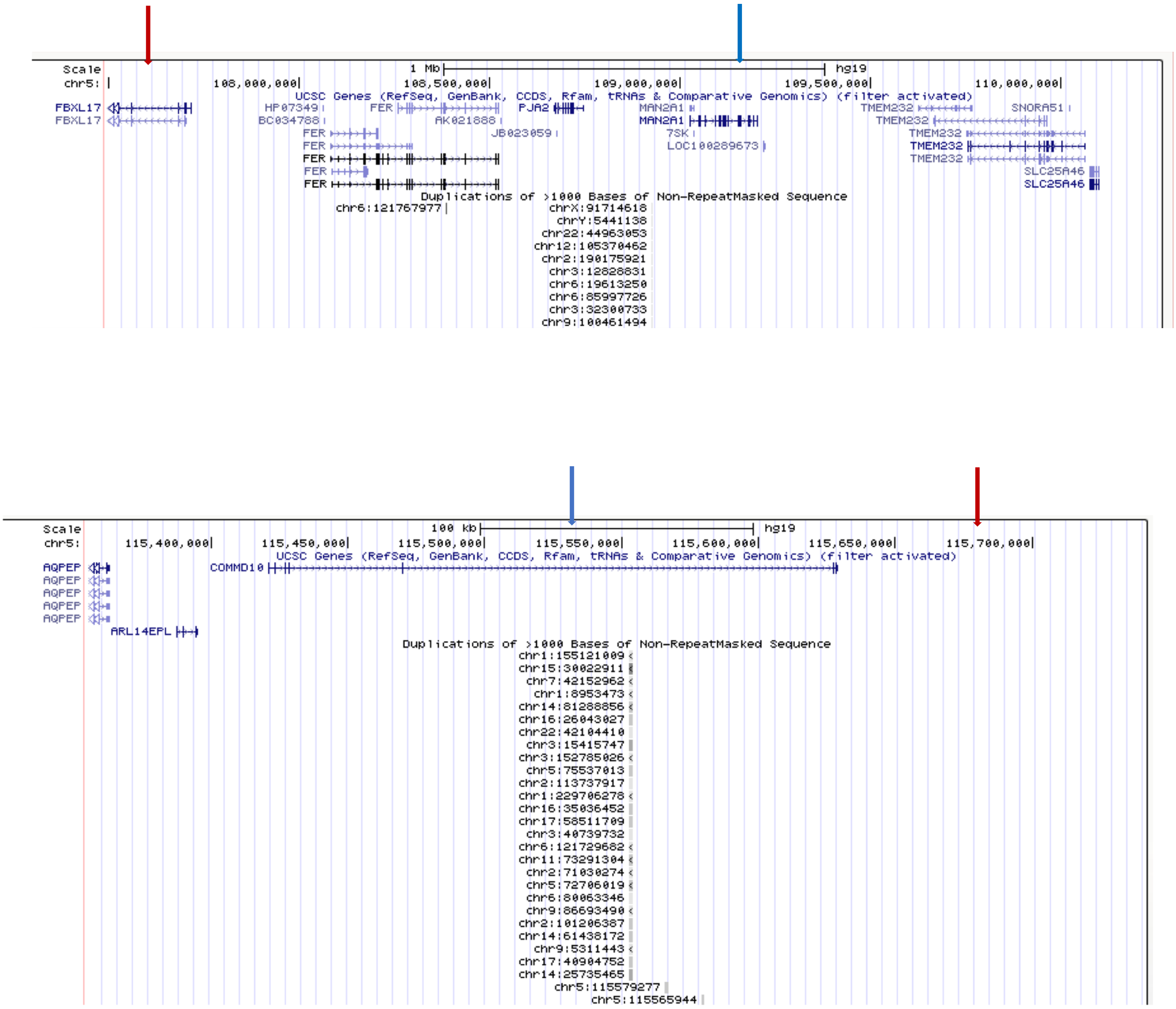

Scale
chr5:
1 Mb
hg19
108,000,000
108,500,000
109,000,000
109,500,000
110,000,000
UCSC Genes (RefSeq, GenBank, CCDS, Rfam, tRNAs & Comparative Genomics) (filter activated)
FBXL17
FBXL17
HP07349
BC034788
FER
FER
FER
FER
FER
FER
AK021888
JB023059
PJA2
MAN2A1
MAN2A1
7SK
LOC100289673
TMEM232
TMEM232
TMEM232
TMEM232
TMEM232
SNORA51
SLC25A46
SLC25A46
Duplications of >1000 Bases of Non-RepeatMasked Sequence
chr6:121767977
chrX:91714618
chrY:5441138
chr22:44963053
chr12:105370462
chr2:190175921
chr3:12828831
chr6:19613250
chr6:85997726
chr3:32300733
chr9:100461494
Scale
chr5:
100 kb
hg19
115,400,000
115,450,000
115,500,000
115,550,000
115,600,000
115,650,000
115,700,000
UCSC Genes (RefSeq, GenBank, CCDS, Rfam, tRNAs & Comparative Genomics) (filter activated)
AQPEP
AQPEP
AQPEP
AQPEP
AQPEP
COMMD10
ARL14EPL
Duplications of >1000 Bases of Non-RepeatMasked Sequence
chr1:155121009
chr15:30022911
chr7:42152962
chr1:8953473
chr14:81288856
chr16:26043027
chr22:42104410
chr3:15415747
chr3:152785026
chr5:75537013
chr2:113737917
chr1:229706278
chr16:35036452
chr17:58511709
chr3:40739732
chr6:121729682
chr11:73291304
chr2:71030274
chr5:72706019
chr6:80063346
chr9:86693490
chr2:101206387
chr14:61438172
chr9:5311443
chr17:40904752
chr14:25735465
chr5:115579277
chr5:115565944

(e)

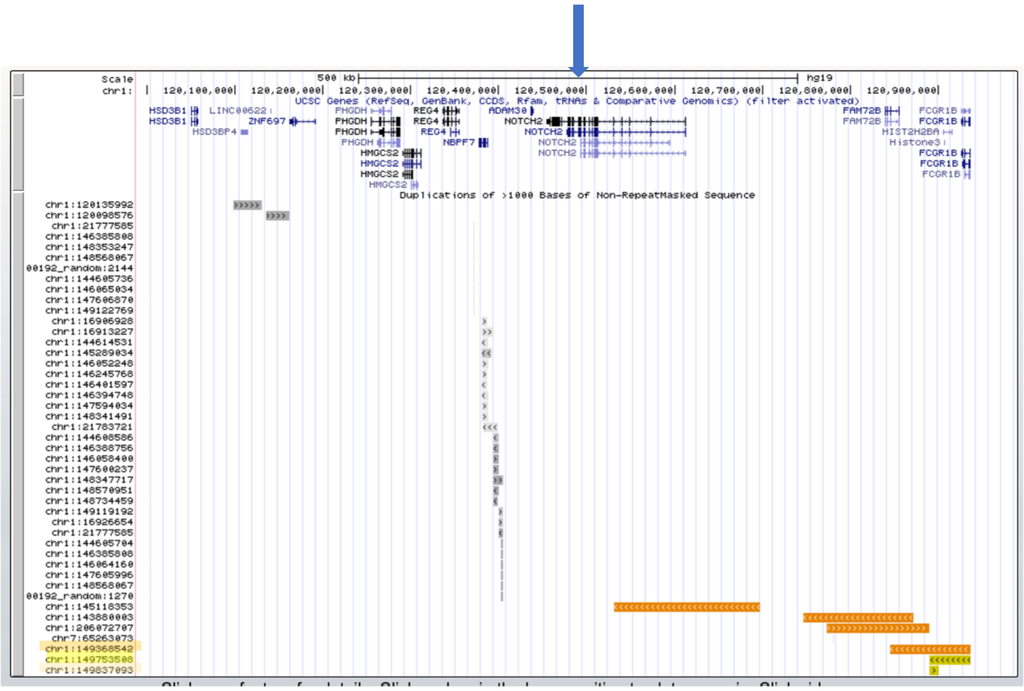
Scale
chr1:
500 kb
hg19
120,100,000 120,200,000 120,300,000 120,400,000 120,500,000 120,600,000 120,700,000 120,800,000 120,900,000
UCSC Genes (RefSeq, GenBank, CCDS, Rfam, tRNAs & Comparative Genomics) (filter activated)
HSD3B1 LINC00622 PHGDH REG4 ADAM30 FAM72B FCGR1B
ZNF697 NOTCH2 HSD3BP4 NBPF7 HIST2H2BA Histone3
HMGCS2
Duplications of >1000 Bases of Non-RepeatMasked Sequence

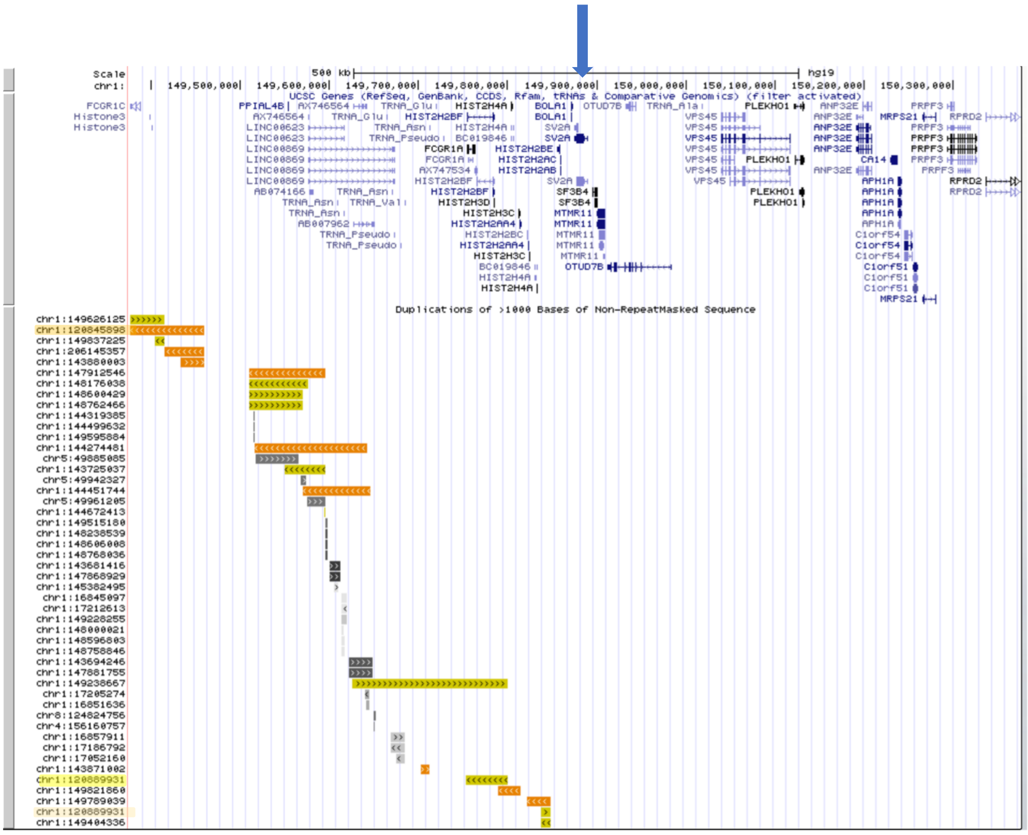
Scale
chr1:
500 kb
hg19
149,500,000 149,600,000 149,700,000 149,800,000 149,900,000 150,000,000 150,100,000 150,200,000 150,300,000
UCSC Genes (RefSeq, GenBank, CCDS, Rfam, tRNAs & Comparative Genomics) (filter activated)
FCGR1C Histone3 PPIAL4B AX746564 TRNA_Glu HIST2H4A BOLA1 OTUD7B TRNA_Ala PLEKHO1 ANP32E PRPF3 MRPS21 RPRD2
LINC00623 LINC00869 TRNA_Asn TRNA_Pseudo BC019846 FCGR1A HIST2H2BE HIST2H2AC HIST2H2AB SV2A VPS45 CA14
AX747534 HIST2H2BF AB074166 TRNA_Val HIST2H3D HIST2H3C HIST2H2AA4 SF3B4 MTMR11 APH1A C1orf54 C1orf51
AB007962 HIST2H2BC OTUD7B
Duplications of >1000 Bases of Non-RepeatMasked Sequence

(f)

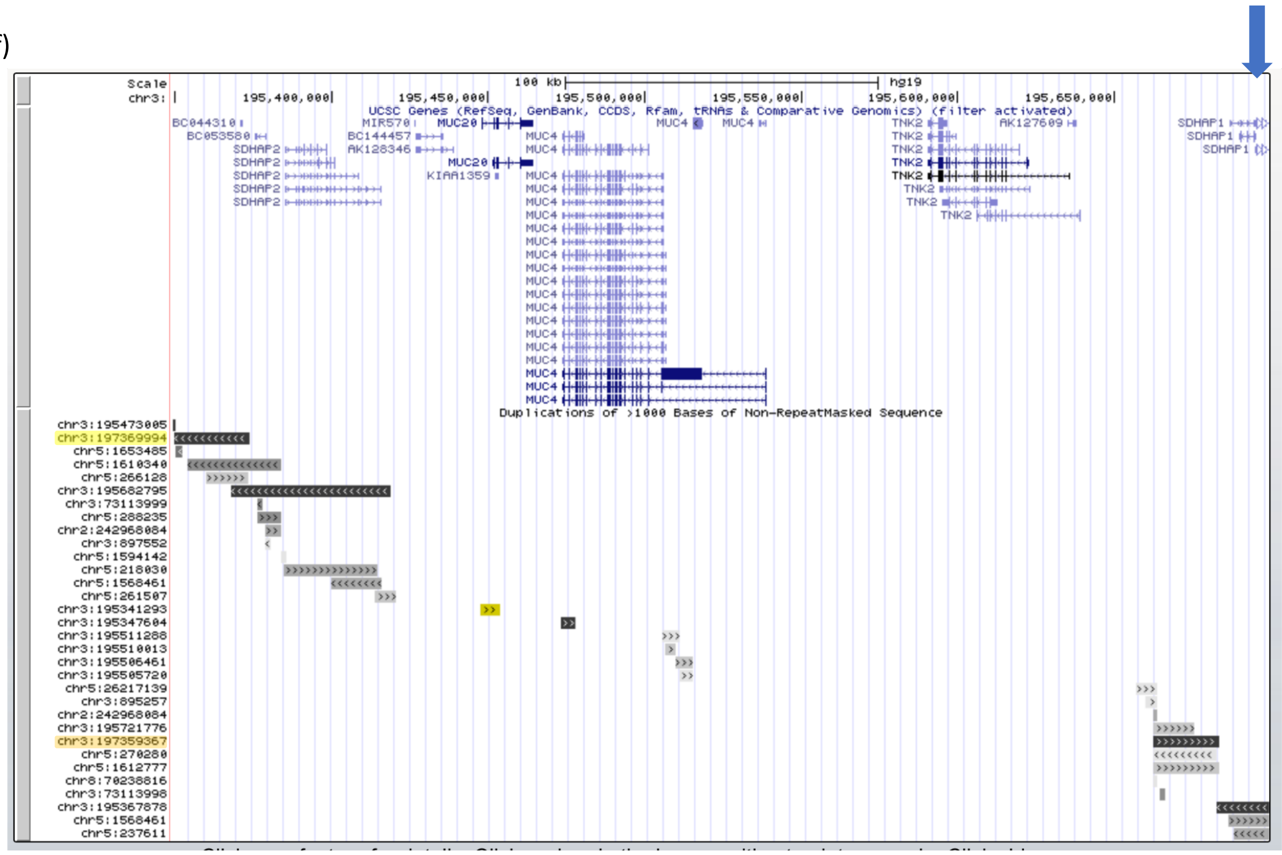
Scale
chr3:
100 kb
hg19
195,400,000
195,450,000
195,500,000
195,550,000
195,600,000
195,650,000
UCSC Genes (RefSeq, GenBank, CCDS, Rfam, tRNAs & Comparative Genomics) (filter activated)
BC044310
BC053580
SDHAP2
BC144457
AK128346
MIR570
MUC20
KIAA1359
MUC4
TNK2
AK127609
SDHAP1
Duplications of >1000 Bases of Non-RepeatMasked Sequence
chr3:195473005
chr3:197369994
chr5:1653485
chr5:1610340
chr5:266128
chr3:195682795
chr3:73113999
chr5:288235
chr2:242968084
chr3:897552
chr5:1594142
chr5:218030
chr5:1568461
chr5:261507
chr3:195341293
chr3:195347604
chr3:195511288
chr3:195510013
chr3:195506461
chr3:195505720
chr5:26217139
chr3:895257
chr2:242968084
chr3:195721776
chr3:197359367
chr5:270280
chr5:1612777
chr8:70238816
chr3:73113998
chr3:195367878
chr5:1568461
chr5:237611

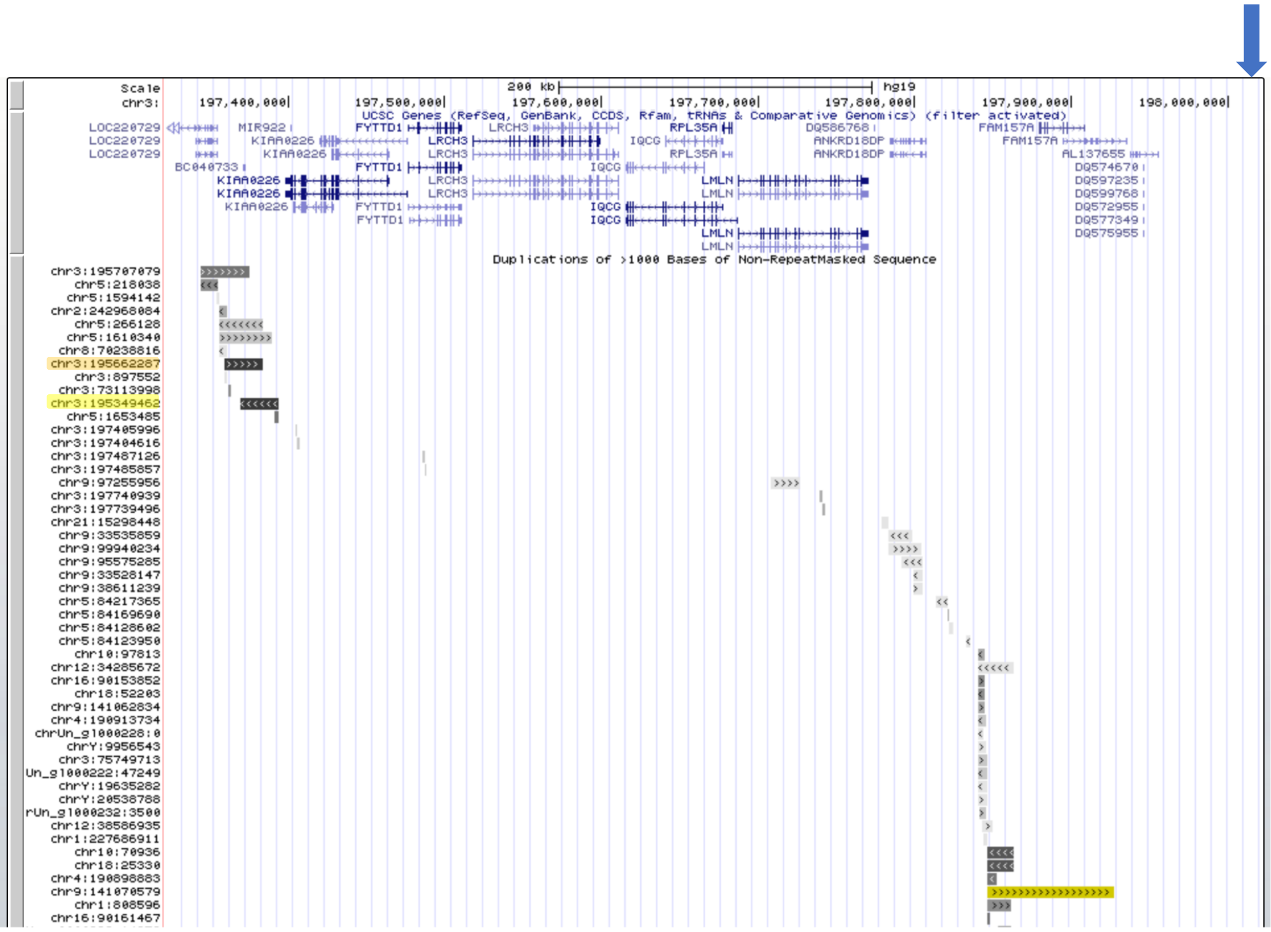
Scale
chr3:
200 kb
hg19
197,400,000
197,500,000
197,600,000
197,700,000
197,800,000
197,900,000
198,000,000
UCSC Genes (RefSeq, GenBank, CCDS, Rfam, tRNAs & Comparative Genomics) (filter activated)
LOC220729
MIR922
KIAA0226
BC040733
FYTTD1
LRCH3
RPL35A
IQCG
LMLN
DQ586768
ANKRD18DP
FAM157A
AL137655
DQ574670
DQ597235
DQ599768
DQ572955
DQ577349
DQ575955
Duplications of >1000 Bases of Non-RepeatMasked Sequence
chr3:195707079
chr5:218038
chr5:1594142
chr2:242968084
chr5:266128
chr5:1610340
chr8:70238816
chr3:195662287
chr3:897552
chr3:73113998
chr3:195349462
chr5:1653485
chr3:197405996
chr3:197404616
chr3:197487126
chr3:197485857
chr9:97255956
chr3:197740939
chr3:197739496
chr21:15298448
chr9:33535859
chr9:99940234
chr9:95575285
chr9:33528147
chr9:38611239
chr5:84217365
chr5:84169690
chr5:84128602
chr5:84123950
chr10:97813
chr12:34285672
chr16:90153852
chr18:52203
chr9:141062834
chr4:190913734
chrUn_gl000228:0
chrY:9956543
chr3:75749713
Un_gl000222:47249
chrY:19635282
chrY:20538788
rUn_gl000232:3500
chr12:38586935
chr1:227686911
chr10:70936
chr18:25330
chr4:190898883
chr9:141070579
chr1:808596
chr16:90161467

Supplementary Fig. S7. Detailed visualisation of boundaries of gains with evidence of shared breakpoints suggesting clonality, and gains possibly arising at segmental duplications (SDs).

(a) Gain in chr17 possibly shared by 3 cells. Top: centromeric boundary, bottom: telomeric boundary region. Boundary is indicated for each cell by an arrow.
(b) Chr9 gain possibly shared by 2 cells. The telomeric boundary is similar to that of a slightly larger DGV reported inversion (chr9:117662397-123567673; nsv7434).
(c) Chr5 gain 1 possibly shared by 2 pontine non-neurons. A paralogous SD flanking the CNV is highlighted. An inversion reported in DGV is shown below (magenta).
(d) Chr5 gain 2 possibly shared by same 2 pontine non-neurons. Both boundary regions contain SDs.
(e) Cell K3. Gain with both boundaries near SD. Three paralogous SDs are highlighted.
(f) Cell H18. Gain with both boundaries near SD, although call extends to telomere. Two paralogous SDs are highlighted. Note only partial view of telomeric SD shown.

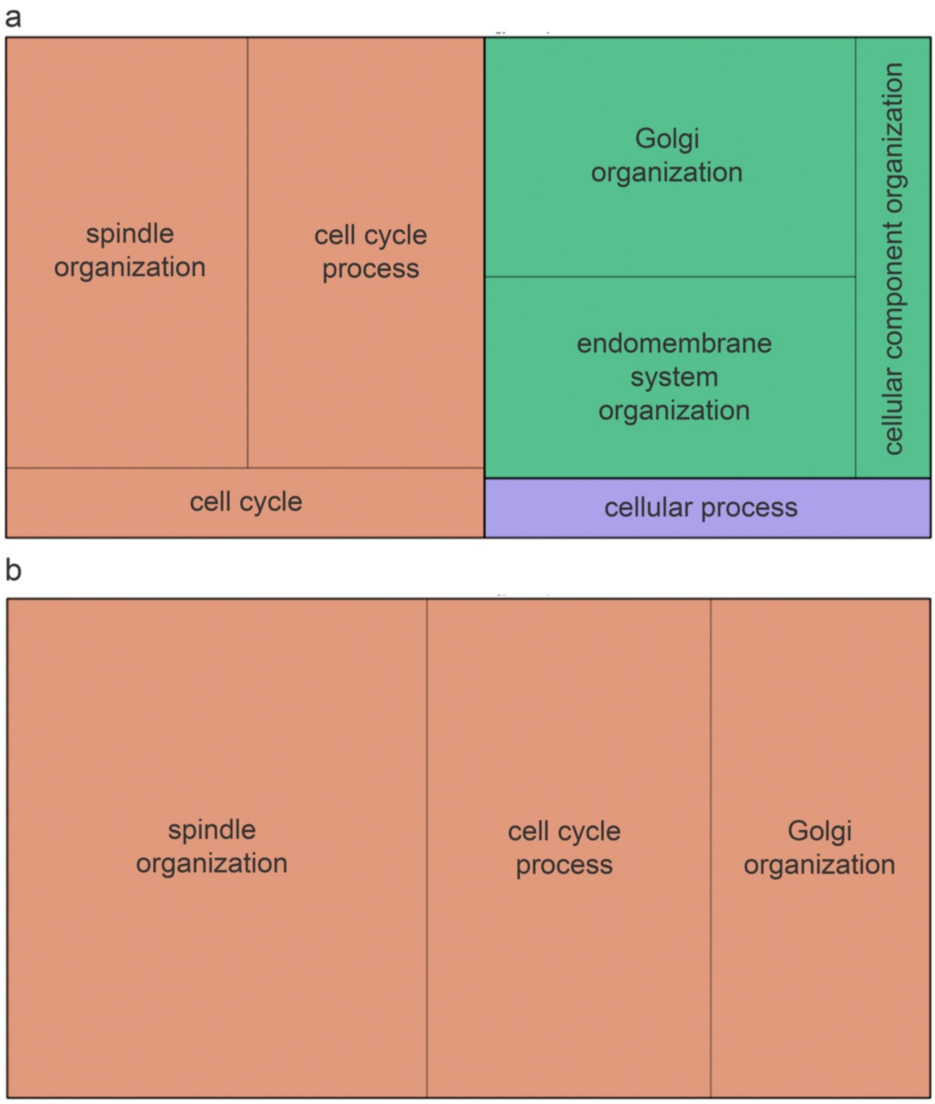

Supplementary Fig. S8. Pathway analysis of neuronal CNVs in each SN separately. a: SND. b: mixed
