# Supplementary material for: Investigation of somatic CNVs in brains of synucleinopathy cases using targeted SNCA analysis and single cell sequencing
Source: Acta Neuropathol Commun. 2019 Dec 23;7:219. doi: 10.1186/s40478-019-0873-5 (PMC6929293; doi:10.1186/s40478-019-0873-5)
Supplement: Supplementary file 3 — Additional file 3: Table S2. Mosaicism % in MSA-SND and mixed MSA in the cingulate cortex and substantia nigra. Table S3. Detailedcorrelation analyses of cingulate cortex and substantia nigra mosaicism. Table S4. Correlation of GCI in the cingulate cortex and sub-cortical region with mosaicism in MSA. Table S5. SNCA mosaicism in occipital cortex, putamen and pons. Table S6 All CNVs which passed filtering. Table S7. Relative over-representation of gene categories in CNVs. Table S8. Enrichment factor in neuronal CNVs in SN. [file 40478_2019_873_MOESM3_ESM.pdf]

Supplementary Tables S2-S7

Supplementary Table S2. **Mosaicism % in MSA-SND and mixed MSA in the cingulate cortex and substantia nigra.** Medians and Mann-Whitney p for comparison of each cell type between the two shown.

		**Mosaicism %**		
Region	Cell type	SND	mixed	p
CC	Neuron	3.07	2.06	0.82
	Non-neuron	1.56	1.70	0.83
SN	NM+	3.57	2.48	0.57
	NM-	2.75	2.33	>0.99

Supplementary Table S3. Detailed correlation analyses of cingulate cortex and substantia nigra mosaicism.

			**Age of onset**		**Age of death**		**Disease duration**	
			r	p	r	p	r	p
MSA	CC	Neuron	0.04	0.903	-0.02	0.936	0.01	0.978
		Non-neuron	-0.07	0.799	0.00	0.977	-0.15	0.601
	SN	NM+	0	>0.99	-0.17	0.624	-0.30	0.390
		NM-	-0.60	0.074	-0.40	0.247	0.40	0.255
PD	CC	Neuron	-0.11	0.623	-0.47	0.019	-0.25	0.240
		Non-neuron	-0.03	0.872	-0.15	0.500	0.02	0.910
Control	CC	Neuron			0.12	0.657		
		Non-neuron			0.46	0.064		

Supplementary Table S4. Correlation of GCI in the cingulate cortex and sub-cortical region with mosaicism in MSA.

	**Cortex**		**Subcortical**	
Cell type	r	p	r	p
Neurons	0.24	0.500	-0.19	0.590
Non-neurons	-0.10	0.793	-0.04	0.904

Supplementary Table S5. ***SNCA*** **mosaicism in occipital cortex, putamen and pons.** The numbers counted, and mosaicism level, are shown for each case, by cell type and brain region.

		**Brain region**					
		**Putamen**		**Occipital**		**Pons**	
**Case**	**Cell type**	**n**	**%**	**n**	**%**	**n**	**%**
**MSA10**	Neuron	101	1.98	124	3.23		
	Non-neuron	117	0.85	114	0.88		
**MSA11**	Neuron					39	7.69
	Non-neuron					103	2.91
**MSA14**	Neuron					31	6.45
	Non-neuron					63	3.18
**MSA15**	Neuron	88	3.41	104	0.96	30	10
	Non-neuron	118	2.54	125	1.60	13	0
**PD6**	Neuron	97	3.09	110	0.91		
	Non-neuron	117	2.56	139	0.72		
**PD8**	Neuron			86	0		
	Non-neuron			110	0.91		
**Control4**	Neuron	108	0				
	Non-neuron	127	0				

Supplementary Table S6. All CNVs which passed filtering.

Cell type: 1=neuron (highlighted), 0=non-neuron. Inclusion: 1=nuclear (in some cases may have also included cytoplasmic in same cell), 2=cytoplasmic, 0=none detected. Gains in green, losses in red. Size in Mb. For possibly clonal CNVs, the co-ordinates are highlighted in the same colour.

CELL	Cell type	Inclusion	co-ordinates			SIZE
D17	1	1	chr1	1998930	6001016	4.00
D17	1	1	chr9	139603642	141213431	1.61
D17	1	1	chr16	89265818	90354753	1.09
F18	0	0	chr17	5372308	13044147	7.67
F30	0	0	chr17	31120575	33278214	2.16
F30	0	0	chr9	121386276	123276584	1.89
F37	1	2	chr1	195440419	201531933	6.09
F37	1	2	chr3	161819105	164572617	2.75
F37	1	2	chr10	26710677	28459488	1.75
F42	0	2	chr16	60592765	67068639	6.48
F42	0	2	chr9	8556699	14489655	5.93
F42	0	2	chr1	19639839	23360178	3.72
F42	0	2	chr2	238598999	239995795	1.40
F53	1	0	chr7	135394704	136773876	1.38
F63	0	2	chrX	24335826	32119406	7.78
F63	0	2	chr12	104801146	110355361	5.55
F63	0	2	chr12	90831552	95233675	4.40
F69	0	0	chr16	76874530	80879178	4.00
F69	0	0	chr10	78716068	81109262	2.39
F69	0	0	chr7	33375650	35451903	2.08
F69	0	0	chr10	72205332	73825201	1.62
F69	0	0	chr17	14438362	16031431	1.59
F69	0	0	chr3	192505544	193876216	1.37
F72	0	2	chr12	125917512	131385950	5.47
F72	0	2	chr16	84704051	87417865	2.71
F72	0	2	chr15	53071073	55558940	2.49
F78	0	0	chrX	10182600	30754561	20.57
F80	0	0	chr16	11997933	18450725	6.45
F80	0	0	chr16	4894165	10252957	5.36
F80	0	0	chr16	22464138	24596023	2.13
F89	1	0	chr22	43392934	49997858	6.60
F89	1	0	chr6	101431246	106673862	5.24
F89	1	0	chr19	28368556	32710608	4.34
F89	1	0	chr22	32919654	36878851	3.96
F89	1	0	chr22	25081506	28174898	3.09
G13	0	0	chr9	111084188	130514878	19.43

G13	0	0	chr9	85133984	95691672	10.56
G13	0	0	chr22	43392934	51304566	7.91
G13	0	0	chr16	84986945	90354753	5.37
G13	0	0	chr17	43577606	47080757	3.50
G13	0	0	chr16	4935399	7747121	2.81
G13	0	0	chr22	25382846	28174898	2.79
G20	0	0	chr1	162233871	166088382	3.85
G20	0	0	chr8	111949911	115205840	3.26
G36	1	2	chr9	117903787	121925025	4.02
G39	0	0	chr1	56723353	59971192	3.25
G47	0	0	chr6	30011756	47050076	17.04
G47	0	0	chr20	4026082	12188971	8.16
G47	0	0	chr16	71142934	73930884	2.79
G49	0	0	chr9	3481291	33456107	29.97
G49	0	0	chr1	100665031	108178290	7.51
G49	0	0	chr1	71124535	76026600	4.90
G54	1	0	chrX	34286664	107957751	73.67
G54	1	0	chr10	75940705	135534747	59.59
G54	1	0	chr4	3579071	61320210	57.74
G54	1	0	chr6	46508075	101431175	54.92
G54	1	0	chr15	1	51088936	51.09
G54	1	0	chr10	30154718	64577506	34.42
G54	1	0	chr2	28733396	61354526	32.62
G54	1	0	chr2	143383709	172620952	29.24
G54	1	0	chr21	1	26979767	26.98
G54	1	0	chr15	75735948	102531392	26.80
G54	1	0	chr2	1	25903532	25.90
G54	1	0	chr2	175415666	198579530	23.16
G54	1	0	chr11	88435059	110854329	22.42
G54	1	0	chr2	100836414	121975326	21.14
G54	1	0	chr2	124726149	134636660	9.91
G54	1	0	chr6	119341812	129233046	9.89
G54	1	0	chr2	77097007	85867838	8.77
G54	1	0	chr15	62195449	70022317	7.83
G54	1	0	chrX	15243488	22026838	6.78
G54	1	0	chrX	149180027	155270560	6.09
G54	1	0	chr15	70022318	75735947	5.71
G54	1	0	chr15	51088937	56746152	5.66
G54	1	0	chr7	23361729	28396813	5.04
G54	1	0	chr22	26563928	31103443	4.54

G54	1	0	chr11	19120286	23521435	4.40
G54	1	0	chr2	208666417	212047501	3.38
G54	1	0	chr15	56746153	60053954	3.31
G54	1	0	chr2	219729922	222964063	3.23
G54	1	0	chr15	60053955	62195448	2.14
G64	1	0	chr14	1	32028173	32.03
G64	1	0	chr4	10898113	16450959	5.55
G64	1	0	chr19	14271942	17402636	3.13
G64	1	0	chr17	55963589	59066071	3.10
G72	0	0	chr12	88673886	106449560	17.78
G72	0	0	chr13	24074740	39564081	15.49
G72	0	0	chr8	91340588	99181153	7.84
G72	0	0	chr12	33606871	40853738	7.25
G72	0	0	chr14	81700562	88747030	7.05
G72	0	0	chr8	133405686	139899211	6.49
G72	0	0	chr18	25208291	30619331	5.41
G72	0	0	chr22	31696440	36878851	5.18
G72	0	0	chr1	44645184	49349371	4.70
G72	0	0	chr12	44180267	48657638	4.48
G72	0	0	chr8	126803186	130663329	3.86
G72	0	0	chr17	44222973	47671469	3.45
G72	0	0	chr8	58244087	61549015	3.30
G72	0	0	chr17	74164066	77054865	2.89
G72	0	0	chr14	75058961	77851050	2.79
G72	0	0	chr11	90146001	92883594	2.74
G74	1	0	chr16	47619557	52524867	4.91
G74	1	0	chr8	37470025	40325008	2.85
G79	1	0	chr11	78451591	81659419	3.21
G86	0	0	chr16	52524868	73389311	20.86
G87	1	0	chr2	55838957	65349001	9.51
G87	1	0	chr18	9293317	14071114	4.78
G87	1	0	chr3	75523310	78386814	2.86
G92	1	0	chr16	6878022	8503416	1.63
H11	1	2	chrX	88395177	93202334	4.81
H11	1	2	chr9	120065847	123276584	3.21
H18	1	0	chr3	195695786	198022430	2.33
H28	0	1	chr2	43206082	53074451	9.87
H32	1	0	chr22	44516162	48926915	4.41
K10	0	0	chr12	122724243	124268799	1.36
K26	0	0	chr2	217526479	219136191	1.61
K27	0	0	chr14	50921239	58497181	7.58
K27	0	0	chr3	158500865	164837036	6.34

K27	0	0	chr3	114039158	119722164	5.68
K27	0	0	chr5	81487219	86714260	5.23
K27	0	0	chr12	125653047	130573668	4.92
K27	0	0	chr3	150374903	154009892	3.63
K27	0	0	chr14	31991032	35084724	3.09
K27	0	0	chr3	173090430	176067429	2.98
K27	0	0	chr20	37043884	39481861	2.44
K27	0	0	chr10	113622747	115243585	1.62
K27	0	0	chr20	56091487	57411419	1.32
K3	1	0	chr1	120489867	149876493	29.39
K3	1	0	chr1	228440457	246279051	17.84
K3	1	0	chr1	156155897	173665573	17.51
K3	1	0	chr4	13605627	25984665	12.38
K3	1	0	chr11	34987795	45163026	10.18
K3	1	0	chr4	81179241	91310527	10.13
K3	1	0	chr1	190564962	200425831	9.86
K3	1	0	chr1	207657043	215719403	8.06
K3	1	0	chr11	22543884	29460309	6.92
K3	1	0	chr6	101153705	105285429	4.13
K3	1	0	chr6	38549878	42357628	3.81
K3	1	0	chr1	47886144	51517067	3.63
K3	1	0	chr10	8907721	12183648	3.28
K3	1	0	chr4	65991857	69045540	3.05
K3	1	0	chr1	102633533	104388033	1.75
K3	1	0	chr10	64030819	65713178	1.68
K31	0	0	chr5	122884417	124248130	1.36
K43	0	0	chr20	5163296	7920389	2.76
K53	0	0	chr12	72707138	109525277	36.82
K53	0	0	chr5	38967846	57402616	18.43
K53	0	0	chr2	80114646	97271570	17.16
K53	0	0	chr11	87155263	101792181	14.64
K53	0	0	chr5	90023239	102743735	12.72
K53	0	0	chr2	56139449	67036007	10.90
K53	0	0	chr12	57258297	67450517	10.19
K53	0	0	chr2	15953289	25903532	9.95
K53	0	0	chr11	121577671	131469532	9.89
K53	0	0	chr12	21405653	31132265	9.73
K53	0	0	chr5	21099152	28940844	7.84
K53	0	0	chr12	113067807	120238957	7.17
K53	0	0	chr11	73533631	79959921	6.43
K53	0	0	chr6	159478885	165818863	6.34
K53	0	0	chr5	109264945	115404722	6.14
K53	0	0	chr5	60465044	66086193	5.62
K53	0	0	chr12	40021121	45576336	5.56
K53	0	0	chr11	105628454	110392991	4.76
K53	0	0	chr5	141813462	146498039	4.68

K53	0	0	chr6	152730077	156041943	3.31
K53	0	0	chr12	1	2672898	2.67
K53	0	0	chr11	59763233	62418974	2.66
K53	0	0	chr11	69381351	71482125	2.10
K59	0	0	chr12	65542447	68818599	3.28
L33	1	0	chr4	77014192	113792449	36.78
L33	1	0	chr3	132276724	160176590	27.90
L33	1	0	chr1	208786233	221144785	12.36
L33	1	0	chr7	55896493	66451132	10.55
L33	1	0	chr1	156727035	164991816	8.26
L33	1	0	chr4	116495408	123926361	7.43
L33	1	0	chr1	79201641	86495506	7.29
L33	1	0	chr4	65430146	71769256	6.34
L33	1	0	chr1	222493872	227294247	4.80
L33	1	0	chr4	38485972	43075945	4.59
L33	1	0	chr4	32215915	35725873	3.51
L33	1	0	chr4	24857649	27113080	2.26
L33	1	0	chr10	69609750	71385347	1.78
L51	1	1	chr2	13483687	15146292	1.66
L62	0	0	chr16	75784146	84704050	8.92
L62	0	0	chr12	102566545	110078086	7.51
L62	0	0	chr16	50393757	53344736	2.95
L62	0	0	chr18	44384415	46549995	2.17
L78	1	0	chr8	142363940	143759855	1.40
L96	0	0	chr3	10320587	11968518	1.65
X11	1	0	chr3	1	170622061	170.62
X11	1	0	chr12	1	109807181	109.81
X11	1	0	chr1	154333116	229295168	74.96
X11	1	0	chr14	49753391	107349540	57.60
X11	1	0	chr11	69656792	122397154	52.74
X11	1	0	chr11	1055662	32446317	31.39
X11	1	0	chr3	170622062	198022430	27.40
X11	1	0	chr17	49068084	72671897	23.60
X11	1	0	chr11	46824447	67177628	20.35
X11	1	0	chr14	23544978	39661652	16.12
X11	1	0	chr16	52260485	67068639	14.81
X11	1	0	chr11	32446318	46824446	14.38
X11	1	0	chr12	121748354	133851895	12.10
X11	1	0	chr18	60943338	72563713	11.62
X11	1	0	chr2	219136192	230684868	11.55
X11	1	0	chr17	27059180	37885663	10.83
X11	1	0	chr8	131797830	142363939	10.57
X11	1	0	chr8	1	9550138	9.55

X11	1	0	chr11	126308155	135006516	8.70
X11	1	0	chr17	72671898	81195210	8.52
X11	1	0	chr1	67064012	75567001	8.50
X11	1	0	chr1	40323492	47886143	7.56
X11	1	0	chr17	42949246	49068083	6.12
X11	1	0	chr17	7085457	12767053	5.68
X11	1	0	chr17	12767054	17641671	4.87
X11	1	0	chr2	65921923	70643732	4.72
X11	1	0	chr1	244574318	249250621	4.68
X11	1	0	chr11	122397155	126308154	3.91
X11	1	0	chr1	1	3686171	3.69
X11	1	0	chr14	45261181	48907640	3.65
X11	1	0	chr17	17641672	21131870	3.49
X11	1	0	chr1	7675495	10638141	2.96
X11	1	0	chr17	37885664	40825112	2.94
X11	1	0	chr11	67177629	69656791	2.48
X11	1	0	chr10	66810017	68456679	1.65
X11	1	0	chr19	11575795	13180319	1.60
X11	1	0	chr11	1	1055661	1.06
X14	1	0	chr5	146776360	164814571	18.04
X14	1	0	chr5	32552736	45778244	13.23
X14	1	0	chr5	128160818	139531603	11.37
X14	1	0	chr5	167787849	176317664	8.53
X14	1	0	chr5	107596167	115691935	8.10
X14	1	0	chr5	119836538	125624145	5.79
X14	1	0	chr10	77085142	82518182	5.43
X14	1	0	chr5	5309902	10197061	4.89
X14	1	0	chr17	44222973	49068083	4.85
X14	1	0	chr5	24589306	29216851	4.63
X14	1	0	chr5	15878114	20403145	4.53
X14	1	0	chr10	95357586	97697737	2.34
X14	1	0	chr2	239995796	242179518	2.18
X14	1	0	chr10	52566310	54463988	1.90
X14	1	0	chr5	66881563	68286581	1.41
X14	1	0	chr5	1	999460	1.00
X16	1	1	chr15	1	68393348	68.39
X16	1	1	chr5	76350348	142355867	66.01
X16	1	1	chr3	135910168	185074777	49.16
X16	1	1	chr18	5200922	43854070	38.65
X16	1	1	chr3	42521467	77087206	34.57
X16	1	1	chr6	41229772	70880716	29.65
X16	1	1	chr9	1	28162618	28.16
X16	1	1	chr5	8015595	31392599	23.38

X16	1	1	chrX	71393805	94585341	23.19
X16	1	1	chrX	133087050	155270560	22.18
X16	1	1	chr6	86287630	108096385	21.81
X16	1	1	chr6	15610126	35153107	19.54
X16	1	1	chr5	43503209	62716924	19.21
X16	1	1	chr9	79196369	97407248	18.21
X16	1	1	chr7	121147414	139340345	18.19
X16	1	1	chr3	97673843	115363492	17.69
X16	1	1	chr14	31111733	47523100	16.41
X16	1	1	chr9	100929975	116004481	15.07
X16	1	1	chr5	154835328	169685339	14.85
X16	1	1	chr5	62716925	76350347	13.63
X16	1	1	chrX	104142134	117758490	13.62
X16	1	1	chr3	9706239	23053055	13.35
X16	1	1	chr3	185074778	198022430	12.95
X16	1	1	chrX	54973496	67702389	12.73
X16	1	1	chr15	90271618	102531392	12.26
X16	1	1	chr3	122058462	134250667	12.19
X16	1	1	chr5	31392600	43503208	12.11
X16	1	1	chrX	117758491	129400007	11.64
X16	1	1	chr15	79786993	90271617	10.48
X16	1	1	chr3	23053056	33268344	10.22
X16	1	1	chr3	1	9706238	9.71
X16	1	1	chr6	139164692	148770396	9.61
X16	1	1	chr12	56325704	65542446	9.22
X16	1	1	chr7	29327046	38240489	8.91
X16	1	1	chr18	59304055	67638750	8.33
X16	1	1	chr5	1	8015594	8.02
X16	1	1	chr5	147051816	154835327	7.78
X16	1	1	chr7	15827369	23540710	7.71
X16	1	1	chr3	79511266	86816478	7.31
X16	1	1	chr22	35719983	42173494	6.45
X16	1	1	chr15	68393349	74514079	6.12
X16	1	1	chr5	175221989	180915260	5.69
X16	1	1	chr5	169685340	175221988	5.54
X16	1	1	chrX	94585342	99951046	5.37
X16	1	1	chr7	143164786	148069846	4.91
X16	1	1	chr20	35332555	40040156	4.71
X16	1	1	chr5	142355868	147051815	4.70
X16	1	1	chr8	105505457	110148311	4.64
X16	1	1	chr12	48977595	53352607	4.38
X16	1	1	chr9	119532595	123836675	4.30
X16	1	1	chr12	100585738	104801145	4.22

X16	1	1	chr6	1	3980925	3.98
X16	1	1	chrX	67702390	71393804	3.69
X16	1	1	chrX	129400008	133087049	3.69
X16	1	1	chr18	74406887	78077248	3.67
X16	1	1	chr20	19874726	23423214	3.55
X16	1	1	chr20	1	3439792	3.44
X16	1	1	chr8	91507692	94804734	3.30
X16	1	1	chr20	47606670	50790966	3.18
X16	1	1	chr14	71419159	74597245	3.18
X16	1	1	chr18	1	2940565	2.94
X16	1	1	chr11	36899014	39641241	2.74
X16	1	1	chr18	55150786	57674598	2.52
X16	1	1	chrX	99951047	102350261	2.40
X16	1	1	chr12	94672482	96973689	2.30
X16	1	1	chrX	102350262	104142133	1.79
X16	1	1	chr22	31103444	32619327	1.52
X16	1	1	chr15	78637409	79786992	1.15
X19	0	2	chr13	1	22654334	22.65
X21	1	1	chr12	132277744	133851895	1.57
X21	1	1	chr4	93291611	94654035	1.36

Supplementary Table S7. Relative over-representation of gene categories in CNVs. MSA SN neurons and non-neurons (this data set), and control cortical neurons (from ref. 71). The results for each annotation module are shown. Significant values (FDR <0.05) are aligned left, and non-significant are aligned right and in italics. Items in blue showed divergence between different cell types, and are discussed in the text.

Annotation		**MSA SN Neurons**	**MSA SN Non-neurons**	**Control cortex Neurons**
**Molecular Function**	Catalytic activity	1.13	*1.05*	*1.02*
	Signalling receptor binding	0.62	*1.08*	*0.88*
**Biological Process**	Spindle assembly	4.12	*0.17*	*2.22*
	Golgi organisation	2.33	*1.06*	*1.70*
	Copper ion	*<0.01*	5.8	4.32
	Cadmium ion	*<0.01*	5.8	4.32
	B cell proliferation	*0.68*	3.96	*0.20*
	Lymphocyte differentiation	*0.40*	4.18	*0.69*
	T cell activation	*0.23*	3.1	*1.31*
	Cellular metal ion homeostasis	*0.81*	2.97	*2.01*
	Peptidyl-serine phosphorylation	*0.63*	2.29	*0.85*
	Innate immune response	0.40	*0.60*	*0.47*
	Humoral immune response	0.32	*0.75*	0.09
	B cell activation	0.25	*0.79*	0.15
	Phagocytosis	0.25	*0.10*	0.21
	Detection of chemical stimulus involved in sensory perception	0.24	*0.25*	*2.63*
	B cell receptor signaling pathway	0.20	<0.01	*0.26*
	Positive regulation of lymphocyte activation	0.11	<0.01	0.05
**Cellular Component**	Golgi cis cisterna	4.73	*<0.01*	*2.67*
	Cis Golgi network	3.3	*0.18*	*2.02*
	Leaflet of membrane bilayer	0.40	*0.76*	0.47
	External side of plasma membrane	0.39	*0.46*	0.36
**Protein class**	Cadherin	*3.97*	5.22	*<0.01*
	MHC antigen	3.89	*0.25*	*0.38*
	Cysteine protease	1.56	*0.95*	*0.90*
	Immunoglobulin receptor superfamily	0.15	*0.62*	*1.46*
	Immunoglobulin	<0.01	*<0.01*	*<0.01*
	Chemokine	<0.01	*1.28*	*0.78*
**Reactome**	Response to metal ion	*0.35*	5.39	*3.70*

Supplementary Table S8. Enrichment factor in neuronal CNVs in SN.

Category	Overall	SND case	Mixed case
**Spindle assembly**	4.17	15.96	4.13
**Spindle organisation**	3.84	14.70	3.81
**Cell cycle process**	2.58	9.19	2.40
**Golgi organisation**	2.36	9.17	2.27
